# Supplementary material for: A multiplexed, confinable CRISPR/Cas9 gene drive can propagate in caged Aedes aegypti populations
Source: Nat Commun. 2024 Jan 25;15:729. doi: 10.1038/s41467-024-44956-2 (PMC10810878; doi:10.1038/s41467-024-44956-2)
Supplement: Supplementary file 1 — Supplementary Information [file 41467_2024_44956_MOESM1_ESM.pdf]

Supplementary Information for

A multiplexed, confinable CRISPR/Cas9 gene drive can propagate in caged *Aedes aegypti* populations

Michelle A.E. Anderson, Estela Gonzalez, Matthew P. Edgington, Joshua X. D. Ang, Deepak-Kumar Purusothaman, Lewis Shackleford, Katherine Nevard, Sebald A. N. Verkuijl, Timothy Harvey-Samuel, Philip T. Leftwich, Kevin Esvelt, Luke Alphey

Email: luke.alphey@york.ac.uk

This PDF file includes:

Supplementary text S1 and S2
Figures S1 to S10
Tables S1 to S12
Legend for Dataset S1

Other supplementary materials for this manuscript include the following:

Dataset S1 Cage trial

Supplementary Text S1. Flanking sequences for *bgcn*-Cas9 line. Flanking PCRs were performed on the *bgcn*-Cas9 transgenic line by digesting the gDNA extracted from 10 individuals with the restriction enzymes BamHI, MspI and NcoI (New England Biolabs). Sequencing of the fragments flanking the *piggyBac* sites was performed from purified the PCR products. Primers used: LA182, LA184, LA186 and LA187 (see Table S11). BLAST search was performed to find potential insertion site in the genome.

***bgcn*-Cas9 line D**

BamHI

TTAAAGGAAATTTATACATTTCATTATGCTGAAGGAATGGAGAGAGATGCTTGTAGATCTATGTAT
TCAAGTAAAATATTTTATAATAGCGTTGATGTGTGGTATTTTTGGGGCCAAGATAGCCGTAGCGG
TAAACGCGCAGCTATTCAGCAAGAAAAAGCTGAGGGTCGTGGGTTCGAATCCCACCGGCCGAG
GATATTTTCGGATTGGAAATTTTCTCGACTTCCCAGGGCATAAAGTATCATCGTACCTGCCACTC
GATATAAATGGTCATTGCTGGCATAGTAAGCTCTCAGTTAATAACTATGGAAGTGCTCATAAGAA
CACTTGAGTTGAGAAGCAAGCTCTATCCCAGTGGGGACGTAACGCCAGAAAGAAGAGAAAGAA
GAAGTGTGGTATTTTGACCTAAAACTTATCACAATGAGCCGAAATTTGTGAGACGAATCTGAATA
CTGATTGCGACAGAAATGAGAACGATACGAATAATTAAGAATACGGCAAGATGCAATTTCATTGC
ATTATTTCCAGAAAGACGATCAAGATTTATCAAAGTATTATTGTACAATGCGAAAGCTGTTTTGGG
AAAGAATTTTTTGGCATCGGTTTGTATCAGCATCATTCACTGCAGTAATGGGAAAATTGCAGGTT
TTCACTGATCAATGCTTAATACGATGGATACTAGCACATCACCCTACTCCACAACATTTGCACGC
ATTTCAATATAACACACAGAATTCCCACGTATTGCCACTGAAAGCCCAAGATTCTCACAGGATTC
CTAGGATTTCCATTTAACCATTCCCACTGAATGCCAGAATCCTCACCATATACCAGAATATCAGTA
TGCACATTTATTTGAATTCCGGGATCCACCGCCCTCCGCACCACGCCCT

Length=893

	Score	E
AaegL5_1 |		
AaegL5_2 |		
AaegL5_3 |		

Sequence found repetitive along *Ae. aegypti* genome

MspI

AGCGTGAAGACGACAGAAAGGGCGTGGTGCGGAGGGCGGGGTGTAGCGTGAAGACGACAGAA
AGGGCGTGGTGCGGAGGGCGGTGCGGTGGGATTCGAACCCACGACCCTCAGCTTTTTCTTGCT
GAATAGCTGCGCGTTTACCGCTACGGCTATCTTGGCCCCAAAAATACCACACATCAACGCTATTA
TAAAATATTTTACTTGAATACATAGATCTACAAGCATCTCTCTCCATTCCTTCAGCATAATGAAATG
TATAAATTTCCTTTAACCCTAGAAAGATAGTCTGCGTAAAATTGACGCATGCATCCTTGAAATATT
GCTCTCTCTTTCTAAATAGCGCGAATCCGTCGCAA

Length=358

	Score	E
AaegL5_1 | organism=Aedes_aegypti_LVP_AGWG | version=AaegL5 | l...	239	6e-60
AaegL5_2 | organism=Aedes_aegypti_LVP_AGWG | version=AaegL5 | l...	214	2e-52
AaegL5_3 | organism=Aedes_aegypti_LVP_AGWG | version=AaegL5 | l...	210	3e-51

Sequence found repetitive along *Ae. aegypti* genome

NcoI

GGTTACTTCTACACCAAAAATGCAATAAAACTACTGTAAATTCAATAAATAACTCTAGTTAAATTAT
CCAATTGCACTTTTCATCGAAATCGCATGGACTAACACGATACGAAAAAACTGCTTAGCGATTGA
CAACAGTCACACCAGAGTGGATCTAAATAAGAGTGGCGTCAATGTCAAAATTGGAACAGCGGCG
AACTGTGAATTCCATACAAATCCGCGTTCCAATGAAGCAGGAGAAAAGGGAAAGGGCGGAGGG
GGGTTCACTCTTGTTTACAGGCACTCTAAGTCACACCACTTTGTGATACAAGTCTCTTCCCGACT
ACCCCCATACTCTACCCCTCTCGGCACGCCATTTTCACTAAAACCTACAAACATAATCACATAAC
GACTTAATTCAAATAAAACCACGCAAAAAAATTACACATCACAAAAATTTATAAAATTTCACCAATG
ACCTCTTTTAATTTAAAAAGCAAACAAAGTTAAATCCATTGTAATGAAAAAAAAAACAAACTTTTGC
CCATCAACGTGTGCGGGAAGCCCCGCGTATAAAAAAAGGTTCCTTTGATTGTGTTGTTTTTCCTG
GCACTGGAACAAATATCCAGATCAATCCCTCAAAAAAAAAAGCCATAATATTTTGAGACCCAATA
AGAAGGGCAAAAATTAAATTTAACCCTAAAAAGAAAATCAATATTTGAAGTACCTTAAAAAAAAAC
AAGCCTTAAATTTACCC

Length=736

	Score	E
AaegL5_3 | organism=Aedes_aegypti_LVP_AGWG | version=AaegL5 | l...	176	1e-40
AaegL5_2 | organism=Aedes_aegypti_LVP_AGWG | version=AaegL5 | l...	174	4e-40
AaegL5_1 | organism=Aedes_aegypti_LVP_AGWG | version=AaegL5 | l...	174	4e-40

Sequence found repetitive along *Ae. aegypti genome*

Supplementary Text S2: Mathematical Modelling

2.1 Fitting Relative Fitness for Individual Gene Drive Constructs

To obtain relative fitness parameter values for individuals heterozygous or homozygous for a single transgenic construct, we first formulate a deterministic population genetics mathematical model that considers an infinite, closed (no migration), panmictic (random mating) population. This model is simulated using the following set of difference equations

$$\begin{aligned} m_{ww}(t) = \frac{1}{2}\Big[& m_{ww}(t-1)f_{ww}(t-1) + \frac{1}{2}m_{ww}(t-1)f_{Xw}(t-1) + \frac{1}{2}m_{Xw}(t-1)f_{ww}(t-1) \\ & + \frac{1}{4}m_{Xw}(t-1)f_{Xw}(t-1)\Big]/\tilde{\Omega}, \end{aligned} \tag{1}$$

$$\begin{aligned} m_{Xw}(t) = \frac{\Omega_{Xw}}{2}\Big[& \frac{1}{2}m_{ww}(t-1)f_{Xw}(t-1) + m_{ww}(t-1)f_{XX}(t-1) + \frac{1}{2}m_{Xw}(t-1)f_{ww}(t-1) \\ & + \frac{1}{2}m_{Xw}(t-1)f_{Xw}(t-1) + \frac{1}{2}m_{Xw}(t-1)f_{XX}(t-1) + m_{XX}(t-1)f_{Xw}(t-1) \\ & + \frac{1}{2}m_{XX}(t-1)f_{Xw}(t-1)\Big]/\tilde{\Omega}, \end{aligned} \tag{2}$$

$$\begin{aligned} m_{XX}(t) = \frac{\Omega_{XX}}{2}\Big[& \frac{1}{4}m_{Xw}(t-1)f_{Xw}(t-1) + \frac{1}{2}m_{Xw}(t-1)f_{XX}(t-1) + \frac{1}{2}m_{XX}f_{Xw}(t-1) \\ & + m_{XX}(t-1)f_{XX}(t-1)\Big]/\tilde{\Omega}, \end{aligned} \tag{3}$$

$$\begin{aligned} f_{ww}(t) = \frac{1}{2}\Big[& m_{ww}(t-1)f_{ww}(t-1) + \frac{1}{2}m_{ww}(t-1)f_{Xw}(t-1) + \frac{1}{2}m_{Xw}(t-1)f_{ww}(t-1) \\ & + \frac{1}{4}m_{Xw}(t-1)f_{Xw}(t-1)\Big]/\tilde{\Omega}, \end{aligned} \tag{4}$$

$$\begin{aligned} f_{Xw}(t) = \frac{\Omega_{Xw}}{2}\Big[& \frac{1}{2}m_{ww}(t-1)f_{Xw}(t-1) + m_{ww}(t-1)f_{XX}(t-1) + \frac{1}{2}m_{Xw}(t-1)f_{ww}(t-1) \\ & + \frac{1}{2}m_{Xw}(t-1)f_{Xw}(t-1) + \frac{1}{2}m_{Xw}(t-1)f_{XX}(t-1) + m_{XX}(t-1)f_{Xw}(t-1) \\ & + \frac{1}{2}m_{XX}(t-1)f_{Xw}(t-1)\Big]/\tilde{\Omega}, \end{aligned} \tag{5}$$

$$\begin{aligned} f_{XX}(t) = \frac{\Omega_{XX}}{2}\Big[& \frac{1}{4}m_{Xw}(t-1)f_{Xw}(t-1) + \frac{1}{2}m_{Xw}(t-1)f_{XX}(t-1) + \frac{1}{2}m_{XX}f_{Xw}(t-1) \\ & + m_{XX}(t-1)f_{XX}(t-1)\Big]/\tilde{\Omega}, \end{aligned} \tag{6}$$

in which *m* and *f* represent males and females of the genotype indicated in their respective subscripts (*ww* denoting wild-type, *Xw* transgene heterozygote and *XX* transgene homozygote) and Ω is the fitness of a given genotype (in subscript) relative to wild-type - assumed equal for both sexes. The overall fitness of the entire population Ω is calculated as the sum of all numerators in equations (1)-(6) and is used as˜ a normalising factor to ensure genotype frequencies fill the entire range from zero to one.

To fit this model to experimental data (main text) we take a simple least squares regression approach. In particular we simultaneously fit relative fitness parameters for transgene heterozygous (Ω_{Xw}) and homozygous (Ω_{XX}) individuals by minimising the total error between two numerical simulations of the mathematical model representing the different single transgene experimental scenarios (fitting to the mean of the two experimental replicates). We model the scenario in which the initial cage setup consists of transgene heterozygous females and wild-type males via initial conditions of the form

$m_{ww}(0) = 0.5, \qquad m_{Xw}(0) = 0, \qquad m_{XX}(0) = 0, \qquad f_{ww}(0) = 0, \qquad f_{Xw}(0) = 0.5, \qquad f_{XX}(0) = 0,$

and the case in which the initial cage consists of transgene heterozygous males and females via initial conditions of the form

$m_{ww}(0) = 0, \qquad m_{Xw}(0) = 0.5, \qquad m_{XX}(0) = 0, \qquad f_{ww}(0) = 0, \qquad f_{Xw}(0) = 0.5, \qquad f_{XX}(0) = 0.$

The mathematical model presented here is simulated across a discretised parameter grid representing the full range of possible parameter vales for the relative fitness of transgene heterozygote and transgene homozygote individuals, with the sum of squared errors between the numerical simulation and mean experimental results calculated at each point. The parameter combination giving the lowest squared error is the deemed to provide the best fit and is thus carried forward into other areas of investigation. Note that to provide a fair comparison to experimental data, mean squared errors are based on transgene carrier frequencies - calculated from our modelling results as the sum of *Xw* and *XX* genotype frequencies. All parameter fitting was performed using Matlab (version R2019a; The MathWorks Inc., Natick, MA).

2.2 Element B

Applying the procedure outlined above to the experimental data for cages containing only transgenic construct B (*bgcn*-Cas9) gives the results in Figure S1. This provides a best fit to the experimental data where B transgene heterozygotes have relative fitness Ω_{Xw}=1 (i.e. no fitness cost) and B transgene homozygotes have relative fitness Ω_{XX}=0.79 (i.e. a fitness cost of 21%). In practice this means there is a significant fitness cost associated with being homozygous for transgenic construct B but no cost to being heterozygous (i.e. they display full wild-type fitness).

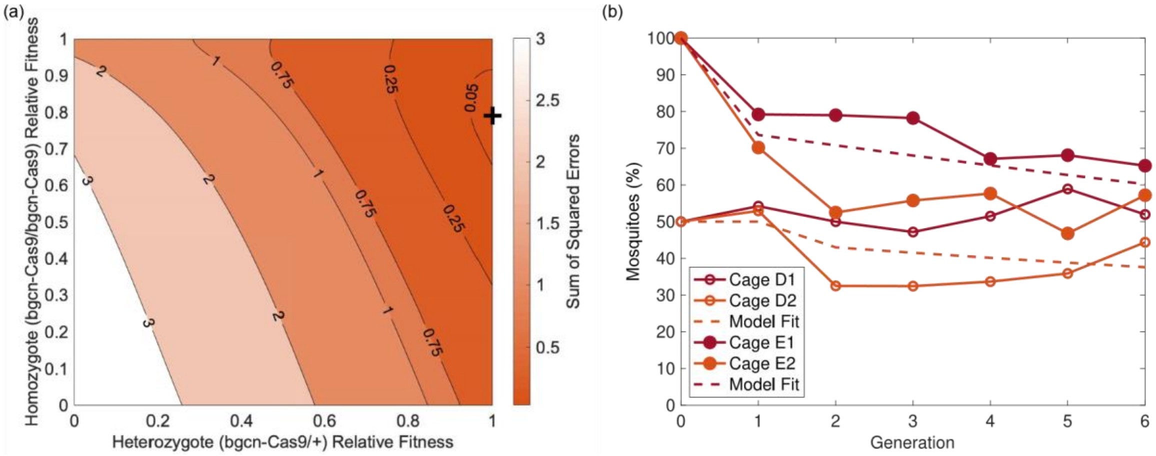

Figure S1: Results of model fitting to experimental data from cage trials containing transgenic construct B (*bgcn*-Cas9) only. (a) Sum of squared errors between numerical simulations and the mean of experimental data for the full range of relative fitness parameters. Here the colour map represents the sum of squared errors with contour lines for certain values included to provide visual clarity. The black cross indicates the parameter combination providing the minimal sum of squared errors. (b) Visualisations of the optimal fit obtained between the mathematical model (dashed lines) and experimental data (solid lines) for the case in which B transgene heterozygous females and wild-type males are used to setup the cage (blue) and where B transgene heterozygote males and females were used to setup the cage (red).

2.3 Element A

A similar procedure was followed for cages containing only transgenic construct A (*kmo*^{sgRNAs}). This gives the results in Figure S2, with the best fit being obtained where A transgene heterozygotes have relative fitness Ω_{Xw}=1 (i.e. no fitness cost) and A transgene homozygotes have relative fitness Ω_{XX}=0.81 (i.e. a fitness cost of 19%). As in the case of the B element, here A construct homozygotes display a fairly significant fitness cost - albeit smaller cost than the B element - while heterozygotes display equal fitness to wild-type individuals.

2.4 Predicting Split-Drive Dynamics

The previous sections used population genetics mathematical models and least squares regression techniques to obtain estimated relative fitness parameters for B+/++, BB/++, ++/A+ and ++/AA genotypes; i.e. those in which only one transgenic construct is present and therefore will not undergo any Cas9-based cleavage nor the resulting homology directed or end-joining repair mechanisms. Since

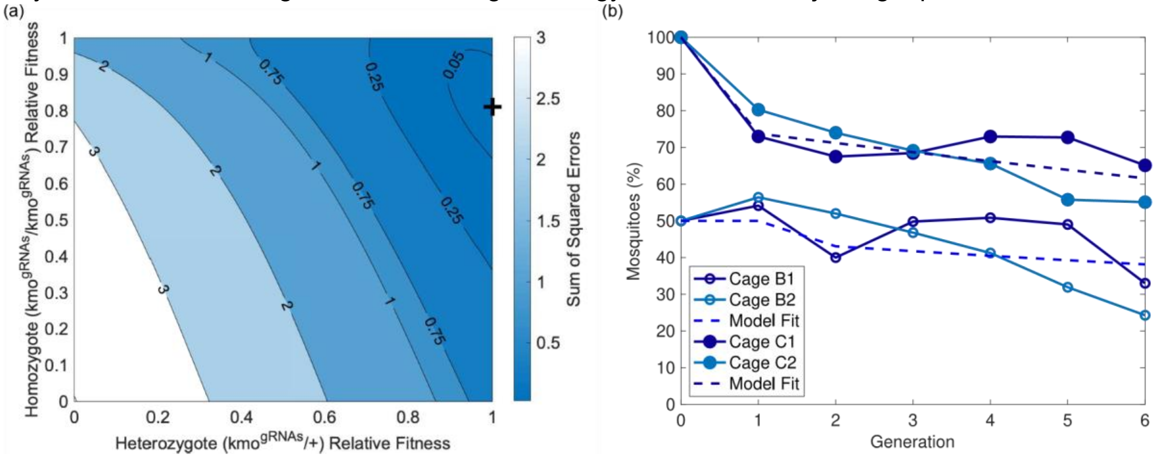

Figure S2: Results of model fitting to experimental data from cage trials containing transgenic construct A (*kmo*^{sgRNAs}) only. (a) Sum of squared errors between numerical simulations and experimental data for all relative fitness parameters considered here. Here the colour map represents the sum of squared errors with contour lines for certain values included to provide visual clarity. The black cross indicates the parameter combination providing the minimal sum of squared errors. (b) Visualisations of the optimal fit obtained between the mathematical model (dashed lines) and experimental data (solid lines) for cases in which B transgene heterozygous females and wild-type males are used to setup the cage (blue) or where B transgene heterozygote males and females were used to setup the cage (red).

experimental work (in the main text) has directly measured homing rate parameters in both males and females, we simply require estimated relative fitness parameters for genotypes in which both the B and A transgenic constructs are present (i.e. B+/A+, B+/AA, BB/A+ and BB/AA). Comparing additive and multiplicative combinations of those relative fitness parameters obtained for B+/++, BB/++, ++/A+ and ++/AA genotypes within the population genetics model outlined in Section 2.1 reveals a slightly smaller sum of squared errors between the mean of experimental data for carrier frequencies of only B or A and those carrying both B and A for a model considering additive fitness costs. This results in relative fitness parameters $\Omega_{B+/A+}$ = 1 (no fitness cost), $\Omega_{B+/AA}$ = 0.81 (19% fitness cost), $\Omega_{BB/A+}$ = 0.79 (21% fitness cost) and $\Omega_{BB/AA}$ = 0.60 (40% fitness cost). In practise the restriction to additive or multiplicative fitness costs means only the relative fitness parameter for the BB/AA genotype displays any difference between the two cases due to the earlier parameter fittings that found zero fitness costs for individuals heterozygous for either one of the transgenic constructs.

Allowing all four of the relative fitness parameters for B+/A+, B+/AA, BB/A+ and BB/AA genotypes to take any value in the range zero to one produces a slightly improved fit to experimental data, however the resulting parameter values did not produce results with any intuitive explanation. Thus we favour the use of an additive combination of relative fitness parameters obtained earlier, producing the results in Figure S3.

2.4.1 Split-Drive Mathematical Model

As in Section 1, we formulate here a deterministic population genetics mathematical model that considers an infinite, closed (no migration), panmictic (random mating) population with a total of nine different genotypes, namely ++/++ (wild-type), ++/A+, ++/AA, B+/++, B+/A+, B+/AA, BB/++,

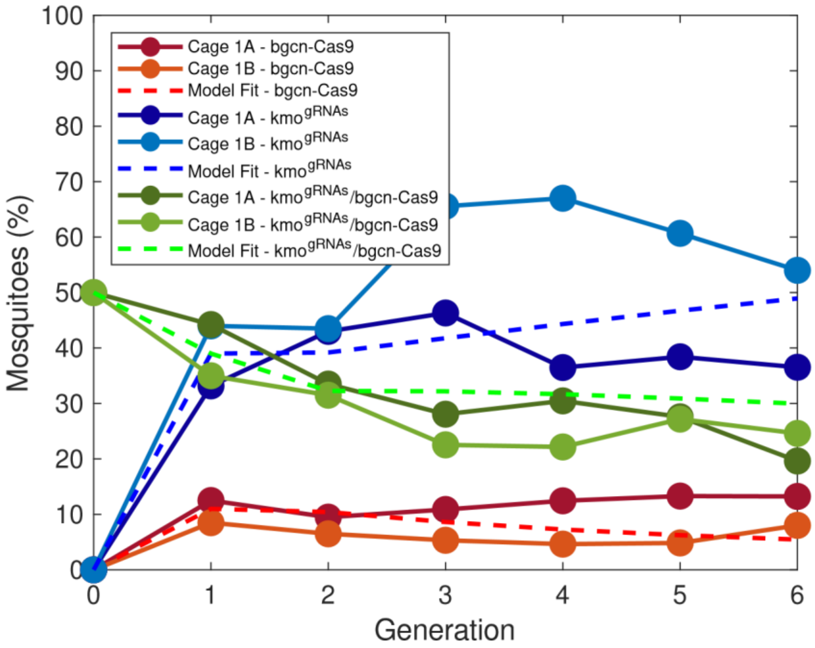

Figure S3: Results of model fitting to experimental data from cage trials containing both split drive transgenic components (*kmo*^{sgRNAs} (A) and *bgcn*-Cas9(B)). Here solid lines show experimental data for the percentage of mosquitoes carrying only the B (red) or A (blue) construct or both the B and A (green) constructs. Dashed lines show results from a population genetics mathematical model considering an additive combination of relative fitness parameters obtained using the single construct mathematical models in Section 1.

BB/A+ and BB/AA. This model is simulated using the following set of difference equations

$$
\begin{aligned}
m_{++/++} = \frac{\Omega_{++/++}}{2} \Big[& m_{++/++}(t-1) f_{++/++}(t-1) + \frac{1}{2} m_{++/++}(t-1) f_{++/A+}(t-1) \\
& + \frac{1}{2} m_{++/++}(t-1) f_{B+/++}(t-1) + \frac{1}{4} m_{++/++}(t-1) f_{B+/A+}(t-1)(1-\Phi_F) \\
& + \frac{1}{2} m_{++/A+}(t-1) f_{++/++}(t-1) + \frac{1}{4} m_{++/A+}(t-1) f_{++/A+}(t-1) \\
& + \frac{1}{4} m_{++/A+}(t-1) f_{B+/++}(t-1) + \frac{1}{8} m_{++/A+}(t-1) f_{B+/A+}(t-1)(1-\Phi_F) \\
& + \frac{1}{2} m_{B+/++}(t-1) f_{++/++}(t-1) + \frac{1}{4} m_{B+/++}(t-1) f_{++/A+}(t-1) \\
& + \frac{1}{4} m_{B+/++}(t-1) f_{B+/++}(t-1) + \frac{1}{8} m_{B+/++}(t-1) f_{B+/A+}(t-1)(1-\Phi_F) \\
& + \frac{1}{4} m_{B+/A+}(t-1)(1-\Phi_M) f_{++/++}(t-1) + \frac{1}{8} m_{B+/A+}(t-1)(1-\Phi_M) f_{++/A+}(t-1) \\
& + \frac{1}{8} m_{B+/A+}(t-1)(1-\Phi_M) f_{B+/++}(t-1) + \frac{1}{16} m_{B+/A+}(t-1)(1-\Phi_M) f_{B+/A+}(t-1)(1-\Phi_F) \Big] / \tilde{\Omega}
\end{aligned}
\tag{7}
$$

$$
\begin{aligned}
m_{++/A+} = \frac{\Omega_{++/A+}}{2} \Big[& \frac{1}{2} m_{++/++}(t-1) f_{++/A+}(t-1) + m_{++/++}(t-1) f_{++/AA}(t-1) \\
& + \frac{1}{4} m_{++/++}(t-1) f_{B+/A+}(t-1)(1-\Phi_F) + \frac{1}{2} m_{++/++}(t-1) f_{B+/AA}(t-1)
\end{aligned}
\tag{8}
$$

$$
\begin{aligned}
&+\frac{1}{2}m_{++/A+}(t-1)f_{++/++}(t-1)+\frac{1}{2}m_{++/A+}(t-1)f_{++/A+}(t-1)\\
&+\frac{1}{2}m_{++/A+}(t-1)f_{++/AA}(t-1)+\frac{1}{4}m_{++/A+}(t-1)f_{B+/++}(t-1)\\
&+\frac{1}{4}m_{++/A+}(t-1)f_{B+/A+}(t-1)(1-\Phi_F)+\frac{1}{4}m_{++/A+}(t-1)f_{B+/AA}(t-1)\\
&+m_{++/AA}(t-1)f_{++/++}(t-1)+\frac{1}{2}m_{++/AA}(t-1)f_{++/A+}(t-1)\\
&+\frac{1}{2}m_{++/AA}(t-1)f_{B+/++}(t-1)+\frac{1}{4}m_{++/AA}(t-1)f_{B+/A+}(t-1)(1-\Phi_F)\\
&+\frac{1}{4}m_{B+/++}(t-1)f_{++/A+}(t-1)+\frac{1}{2}m_{B+/++}(t-1)f_{++/AA}(t-1)\\
&+\frac{1}{8}m_{B+/++}(t-1)f_{B+/A+}(t-1)(1-\Phi_F)+\frac{1}{4}m_{B+/++}(t-1)f_{B+/AA}(t-1)\\
&+\frac{1}{4}m_{B+/A+}(t-1)(1-\Phi_M)f_{++/++}(t-1)+\frac{1}{4}m_{B+/A+}(t-1)(1-\Phi_M)f_{++/A+}(t-1)\\
&+\frac{1}{4}m_{B+/A+}(t-1)(1-\Phi_M)f_{++/AA}(t-1)+\frac{1}{8}m_{B+/A+}(t-1)(1-\Phi_M)f_{B+/++}(t-1)\\
&+\frac{1}{8}m_{B+/A+}(t-1)(1-\Phi_M)f_{B+/A+}(t-1)(1-\Phi_F)+\frac{1}{8}m_{B+/A+}(t-1)(1-\Phi_M)f_{B+/AA}(t-1)\\
&+\frac{1}{2}m_{B+/AA}(t-1)f_{++/++}(t-1)+\frac{1}{4}m_{B+/AA}(t-1)f_{++/A+}(t-1)\\
&+\frac{1}{4}m_{B+/AA}(t-1)f_{B+/++}(t-1)+\frac{1}{8}m_{B+/AA}(t-1)f_{B+/A+}(t-1)(1-\Phi_F)\\
&+\frac{1}{2}m_{++/++}(t-1)f_{B+/A+}(t-1)\Phi_F+\frac{1}{4}m_{++/A+}(t-1)f_{B+/A+}(t-1)\Phi_F\\
&+\frac{1}{4}m_{B+/++}(t-1)f_{B+/A+}(t-1)\Phi_F+\frac{1}{8}m_{B+/A+}(t-1)f_{B+/A+}(t-1)\Phi_F(1-\Phi_M)\\
&+\frac{1}{2}m_{B+/A+}(t-1)f_{++/++}(t-1)\Phi_M+\frac{1}{4}m_{B+/A+}(t-1)f_{++/A+}(t-1)\Phi_M\\
&+\frac{1}{4}m_{B+/A+}(t-1)f_{B+/++}(t-1)\Phi_M+\frac{1}{8}m_{B+/A+}(t-1)f_{B+/A+}(t-1)\Phi_M(1-\Phi_F)\\
&+\left.\frac{1}{8}(1-\Phi_M)m_{B+/A+}(t-1)\Phi_F f_{BB/A+}(t-1)\right]/\tilde{\Omega},
\end{aligned}
$$

$$
\begin{aligned}
m_{++/AA}=\frac{\Omega_{++/AA}}{2}&\left[\frac{1}{4}m_{++/A+}(t-1)f_{++/A+}(t-1)+\frac{1}{2}m_{++/A+}(t-1)f_{++/AA}(t-1)\right. \qquad (9)\\
&+\frac{1}{8}m_{++/A+}(t-1)f_{B+/A+}(t-1)(1-\Phi_F)+\frac{1}{4}m_{++/A+}(t-1)f_{B+/AA}(t-1)\\
&+\frac{1}{2}m_{++/AA}(t-1)f_{++/A+}(t-1)+m_{++/AA}(t-1)f_{++/AA}(t-1)\\
&+\frac{1}{4}m_{++/AA}(t-1)f_{B+/A+}(t-1)(1-\Phi_F)+\frac{1}{2}m_{++/AA}(t-1)f_{B+/AA}(t-1)\\
&+\frac{1}{8}m_{B+/A+}(t-1)(1-\Phi_M)f_{++/A+}(t-1)+\frac{1}{4}m_{B+/A+}(t-1)(1-\Phi_M)f_{++/AA}(t-1)\\
&+\frac{1}{16}m_{B+/A+}(t-1)(1-\Phi_M)f_{B+/A+}(t-1)(1-\Phi_F)+\frac{1}{8}m_{B+/A+}(t-1)(1-\Phi_M)f_{B+/AA}(t-1)\\
&+\frac{1}{4}m_{B+/AA}(t-1)f_{++/A+}(t-1)+\frac{1}{2}m_{B+/AA}(t-1)f_{++/AA}(t-1)\\
&+\frac{1}{8}m_{B+/AA}(t-1)f_{B+/A+}(t-1)(1-\Phi_F)+\frac{1}{4}m_{B+/AA}(t-1)f_{B+/AA}(t-1)\\
&+\frac{1}{4}m_{++/A+}(t-1)f_{B+/A+}(t-1)\Phi_F+\frac{1}{2}m_{++/AA}(t-1)f_{B+/A+}(t-1)\Phi_F\\
&+\frac{1}{8}m_{B+/A+}(t-1)f_{B+/A+}(t-1)\Phi_F(1-\Phi_M)+\frac{1}{4}m_{B+/AA}(t-1)f_{B+/A+}(t-1)\Phi_F\\
&+\frac{1}{4}m_{B+/A+}(t-1)f_{++/A+}(t-1)\Phi_M+\frac{1}{2}m_{B+/A+}(t-1)f_{++/AA}(t-1)\Phi_M\\
&+\frac{1}{8}m_{B+/A+}(t-1)f_{B+/A+}(t-1)\Phi_M(1-\Phi_F)+\frac{1}{4}m_{B+/A+}(t-1)f_{B+/AA}(t-1)\Phi_M
\end{aligned}
$$

$$
\begin{aligned}
&+\frac{1}{4}m_{B+/A+}(t-1)f_{B+/A+}(t-1)\Phi_M\Phi_F+\frac{1}{8}(1-\Phi_M)m_{B+/A+}(t-1)\Phi_F f_{BB/A+}(t-1)\Big]/\tilde{\Omega},\\
m_{B+/++} = \frac{\Omega_{B+/++}}{2}&\Big[\frac{1}{2}m_{++/++}(t-1)f_{B+/++}(t-1)+\frac{1}{4}m_{++/++}(t-1)f_{B+/A+}(t-1)(1-\Phi_F) \qquad (10)\\
&+m_{++/++}(t-1)f_{BB/++}(t-1)+\frac{1}{2}m_{++/++}(t-1)f_{BB/A+}(t-1)(1-\Phi_F)\\
&+\frac{1}{4}m_{++/A+}(t-1)f_{B+/++}(t-1)+\frac{1}{8}m_{++/A+}(t-1)f_{B+/A+}(t-1)(1-\Phi_F)\\
&+\frac{1}{2}m_{++/A+}(t-1)f_{BB/++}(t-1)+\frac{1}{4}m_{++/A+}(t-1)f_{BB/A+}(t-1)(1-\Phi_F)\\
&+\frac{1}{2}m_{B+/++}(t-1)f_{++/++}(t-1)+\frac{1}{4}m_{B+/++}(t-1)f_{++/A+}(t-1)\\
&+\frac{1}{2}m_{B+/++}(t-1)f_{B+/++}(t-1)+\frac{1}{4}m_{B+/++}(t-1)f_{B+/A+}(t-1)(1-\Phi_F)\\
&+\frac{1}{2}m_{B+/++}(t-1)f_{BB/++}(t-1)+\frac{1}{4}m_{B+/++}(t-1)f_{BB/A+}(t-1)(1-\Phi_F)\\
&+\frac{1}{4}m_{B+/A+}(t-1)(1-\Phi_M)f_{++/++}(t-1)+\frac{1}{8}m_{B+/A+}(t-1)(1-\Phi_M)f_{++/A+}(t-1)\\
&+\frac{1}{4}m_{B+/A+}(t-1)(1-\Phi_M)f_{B+/++}(t-1)+\frac{1}{8}m_{B+/A+}(t-1)(1-\Phi_M)f_{B+/A+}(t-1)(1-\Phi_F)\\
&+\frac{1}{4}m_{B+/A+}(t-1)(1-\Phi_M)f_{BB/++}(t-1)+\frac{1}{8}m_{B+/A+}(t-1)(1-\Phi_M)f_{BB/A+}(t-1)(1-\Phi_F)\\
&+m_{BB/++}(t-1)f_{++/++}(t-1)+\frac{1}{2}m_{BB/++}(t-1)f_{++/A+}(t-1)\\
&+\frac{1}{2}m_{BB/++}(t-1)f_{B+/++}(t-1)+\frac{1}{4}m_{BB/++}(t-1)f_{B+/A+}(t-1)(1-\Phi_F)\\
&+\frac{1}{2}m_{BB/A+}(t-1)(1-\Phi_M)f_{++/++}(t-1)+\frac{1}{4}m_{BB/A+}(t-1)(1-\Phi_M)f_{++/A+}(t-1)\\
&+\frac{1}{4}m_{BB/A+}(t-1)(1-\Phi_M)f_{B+/++}(t-1)+\frac{1}{8}m_{BB/A+}(t-1)(1-\Phi_M)f_{B+/A+}(t-1)(1-\Phi_F)\Big]/\tilde{\Omega}\\
m_{B+/A+} = \frac{\Omega_{B+/A+}}{2}&\Big[\frac{1}{4}m_{++/++}(t-1)f_{B+/A+}(t-1)(1-\Phi_F)+\frac{1}{2}m_{++/++}(t-1)f_{B+/AA}(t-1) \qquad (11)\\
&+\frac{1}{2}m_{++/++}(t-1)f_{BB/A+}(t-1)(1-\Phi_F)+m_{++/++}(t-1)f_{BB/AA}(t-1)\\
&+\frac{1}{4}m_{++/A+}(t-1)f_{B+/++}(t-1)+\frac{1}{4}m_{++/A+}(t-1)f_{B+/A+}(t-1)(1-\Phi_F)\\
&+\frac{1}{4}m_{++/A+}(t-1)f_{B+/AA}(t-1)+\frac{1}{2}m_{++/A+}(t-1)f_{BB/++}(t-1)\\
&+\frac{1}{2}m_{++/A+}(t-1)f_{BB/A+}(t-1)(1-\Phi_F)+\frac{1}{2}m_{++/A+}(t-1)f_{BB/AA}(t-1)\\
&+\frac{1}{2}m_{++/AA}(t-1)f_{B+/++}(t-1)+\frac{1}{4}m_{++/AA}(t-1)f_{B+/A+}(t-1)(1-\Phi_F)\\
&+m_{++/AA}(t-1)f_{BB/++}(t-1)+\frac{1}{2}m_{++/AA}(t-1)f_{BB/A+}(t-1)(1-\Phi_F)\\
&+\frac{1}{4}m_{B+/++}(t-1)f_{++/A+}(t-1)+\frac{1}{2}m_{B+/++}(t-1)f_{++/AA}(t-1)\\
&+\frac{1}{4}m_{B+/++}(t-1)f_{B+/A+}(t-1)(1-\Phi_F)+\frac{1}{2}m_{B+/++}(t-1)f_{B+/AA}(t-1)\\
&+\frac{1}{4}m_{B+/++}(t-1)f_{BB/A+}(t-1)(1-\Phi_F)+\frac{1}{2}m_{B+/++}(t-1)f_{BB/AA}(t-1)\\
&+\frac{1}{4}m_{B+/A+}(t-1)(1-\Phi_M)f_{++/++}(t-1)+\frac{1}{4}m_{B+/A+}(t-1)(1-\Phi_M)f_{++/A+}(t-1)\\
&+\frac{1}{4}m_{B+/A+}(t-1)(1-\Phi_M)f_{++/AA}(t-1)+\frac{1}{4}m_{B+/A+}(t-1)(1-\Phi_M)f_{B+/++}(t-1)\\
&+\frac{1}{4}m_{B+/A+}(t-1)(1-\Phi_M)f_{B+/A+}(t-1)(1-\Phi_F)+\frac{1}{4}m_{B+/A+}(t-1)(1-\Phi_M)f_{B+/AA}(t-1)
\end{aligned}
$$

$$
\begin{aligned}
&+\frac{1}{4}m_{B+/A+}(t-1)(1-\Phi_M)f_{BB/++}(t-1)+\frac{1}{4}m_{B+/A+}(t-1)(1-\Phi_M)f_{BB/A+}(t-1)(1-\Phi_F)\\
&+\frac{1}{4}m_{B+/A+}(t-1)(1-\Phi_M)f_{BB/AA}(t-1)+\frac{1}{2}m_{B+/AA}(t-1)f_{++/++}(t-1)\\
&+\frac{1}{4}m_{B+/AA}(t-1)f_{++/A+}(t-1)+\frac{1}{2}m_{B+/AA}(t-1)f_{B+/++}(t-1)\\
&+\frac{1}{4}m_{B+/AA}(t-1)f_{B+/A+}(t-1)(1-\Phi_F)+\frac{1}{2}m_{B+/AA}(t-1)f_{BB/++}(t-1)\\
&+\frac{1}{4}m_{B+/AA}(t-1)f_{BB/A+}(t-1)(1-\Phi_F)+\frac{1}{2}m_{BB/++}(t-1)f_{++/A+}(t-1)\\
&+m_{BB/++}(t-1)f_{++/AA}(t-1)+\frac{1}{4}m_{BB/++}(t-1)f_{B+/A+}(t-1)(1-\Phi_F)\\
&+\frac{1}{2}m_{BB/++}(t-1)f_{B+/AA}(t-1)+\frac{1}{2}m_{BB/A+}(t-1)(1-\Phi_M)f_{++/++}(t-1)\\
&+\frac{1}{2}m_{BB/A+}(t-1)(1-\Phi_M)f_{++/A+}(t-1)+\frac{1}{2}m_{BB/A+}(t-1)(1-\Phi_M)f_{++/AA}(t-1)\\
&+\frac{1}{4}m_{BB/A+}(t-1)(1-\Phi_M)f_{B+/++}(t-1)+\frac{1}{4}m_{BB/A+}(t-1)(1-\Phi_M)f_{B+/A+}(t-1)(1-\Phi_F)\\
&+\frac{1}{4}m_{BB/A+}(t-1)(1-\Phi_M)f_{B+/AA}(t-1)+m_{BB/AA}(t-1)f_{++/++}(t-1)\\
&+\frac{1}{2}m_{BB/AA}(t-1)f_{++/A+}(t-1)+\frac{1}{2}m_{BB/AA}(t-1)f_{B+/++}(t-1)\\
&+\frac{1}{4}m_{BB/AA}(t-1)f_{B+/A+}(t-1)(1-\Phi_F)+\frac{1}{2}m_{++/++}(t-1)f_{B+/A+}(t-1)\Phi_F\\
&+m_{++/++}(t-1)f_{BB/A+}(t-1)\Phi_F+\frac{1}{4}m_{++/A+}(t-1)f_{B+/A+}(t-1)\Phi_F\\
&+\frac{1}{2}m_{++/A+}(t-1)f_{BB/A+}(t-1)\Phi_F+\frac{1}{2}m_{B+/++}(t-1)f_{B+/A+}(t-1)\Phi_F\\
&+\frac{1}{2}m_{B+/++}(t-1)f_{BB/A+}(t-1)\Phi_F+\frac{1}{4}m_{B+/A+}(t-1)f_{B+/A+}(t-1)\Phi_F(1-\Phi_M)\\
&+\frac{1}{4}m_{B+/A+}(t-1)f_{BB/A+}(t-1)\Phi_F(1-\Phi_M)+\frac{1}{2}m_{BB/++}(t-1)f_{B+/A+}(t-1)\Phi_F\\
&+\frac{1}{4}m_{BB/A+}(t-1)f_{B+/A+}(t-1)\Phi_F(1-\Phi_M)+\frac{1}{2}m_{B+/A+}(t-1)f_{++/++}(t-1)\Phi_M\\
&+\frac{1}{4}m_{B+/A+}(t-1)f_{++/A+}(t-1)\Phi_M+\frac{1}{2}m_{B+/A+}(t-1)f_{B+/++}(t-1)\Phi_M\\
&+\frac{1}{4}m_{B+/A+}(t-1)f_{B+/A+}(t-1)\Phi_M(1-\Phi_F)+\frac{1}{2}m_{B+/A+}(t-1)f_{BB/++}(t-1)\Phi_M\\
&+\frac{1}{4}m_{B+/A+}(t-1)f_{BB/A+}(t-1)\Phi_M(1-\Phi_F)+m_{BB/A+}(t-1)f_{++/++}(t-1)\Phi_M\\
&+\frac{1}{2}m_{BB/A+}(t-1)f_{++/A+}(t-1)\Phi_M+\frac{1}{2}m_{BB/A+}(t-1)f_{B+/++}(t-1)\Phi_M\\
&\left.+\frac{1}{4}m_{BB/A+}(t-1)f_{B+/A+}(t-1)\Phi_M(1-\Phi_F)\right]/\tilde{\Omega},
\end{aligned}
$$

$$
\begin{aligned}
m_{B+/AA}=\frac{\Omega_{B+/AA}}{2}&\left[\frac{1}{8}m_{++/A+}(t-1)f_{B+/A+}(t-1)(1-\Phi_F)+\frac{1}{4}m_{++/A+}(t-1)f_{B+/AA}(t-1)\right. \qquad (12)\\
&+\frac{1}{4}m_{++/A+}(t-1)f_{BB/A+}(t-1)(1-\Phi_F)+\frac{1}{2}m_{++/A+}(t-1)f_{BB/AA}(t-1)\\
&+\frac{1}{4}m_{++/AA}(t-1)f_{B+/A+}(t-1)(1-\Phi_F)+\frac{1}{2}m_{++/AA}(t-1)f_{B+/AA}(t-1)\\
&+\frac{1}{2}m_{++/AA}(t-1)f_{BB/A+}(t-1)(1-\Phi_F)+m_{++/AA}(t-1)f_{BB/AA}(t-1)\\
&+\frac{1}{8}m_{B+/A+}(t-1)(1-\Phi_M)f_{++/A+}(t-1)+\frac{1}{4}m_{B+/A+}(t-1)(1-\Phi_M)f_{++/AA}(t-1)\\
&+\frac{1}{8}m_{B+/A+}(t-1)(1-\Phi_M)f_{B+/A+}(t-1)(1-\Phi_F)+\frac{1}{4}m_{B+/A+}(t-1)(1-\Phi_M)f_{B+/AA}(t-1)
\end{aligned}
$$

$$
\begin{aligned}
&+\frac{1}{8}m_{B+/A+}(t-1)(1-\Phi_M)f_{BB/A+}(t-1)(1-\Phi_F)+\frac{1}{4}m_{B+/A+}(t-1)(1-\Phi_M)f_{BB/AA}(t-1)\\
&+\frac{1}{4}m_{B+/AA}(t-1)f_{++/A+}(t-1)+\frac{1}{2}m_{B+/AA}(t-1)f_{++/AA}(t-1)\\
&+\frac{1}{4}m_{B+/AA}(t-1)f_{B+/A+}(t-1)(1-\Phi_F)+\frac{1}{2}m_{B+/AA}(t-1)f_{B+/AA}(t-1)\\
&+\frac{1}{4}m_{B+/AA}(t-1)f_{BB/A+}(t-1)(1-\Phi_F)+\frac{1}{2}m_{B+/AA}(t-1)f_{BB/AA}(t-1)\\
&+\frac{1}{4}m_{BB/A+}(t-1)(1-\Phi_M)f_{++/A+}(t-1)+\frac{1}{2}m_{BB/A+}(t-1)(1-\Phi_M)f_{++/AA}(t-1)\\
&+\frac{1}{8}m_{BB/A+}(t-1)(1-\Phi_M)f_{B+/A+}(t-1)(1-\Phi_F)+\frac{1}{4}m_{BB/A+}(t-1)(1-\Phi_M)f_{B+/AA}(t-1)\\
&+\frac{1}{2}m_{BB/AA}(t-1)f_{++/A+}(t-1)+m_{BB/AA}(t-1)f_{++/AA}(t-1)\\
&+\frac{1}{4}m_{BB/AA}(t-1)f_{B+/A+}(t-1)(1-\Phi_F)+\frac{1}{2}m_{BB/AA}(t-1)f_{B+/AA}(t-1)\\
&+\frac{1}{4}m_{++/A+}(t-1)f_{B+/A+}(t-1)\Phi_F+\frac{1}{2}m_{++/A+}(t-1)f_{BB/A+}(t-1)\Phi_F\\
&+\frac{1}{2}m_{++/AA}(t-1)f_{B+/A+}(t-1)\Phi_F+m_{++/AA}(t-1)f_{BB/A+}(t-1)\Phi_F\\
&+\frac{1}{4}m_{B+/A+}(t-1)f_{B+/A+}(t-1)\Phi_F(1-\Phi_M)+\frac{1}{4}m_{B+/A+}(t-1)f_{BB/A+}(t-1)\Phi_F(1-\Phi_M)\\
&+\frac{1}{2}m_{B+/AA}(t-1)f_{B+/A+}(t-1)\Phi_F+\frac{1}{2}m_{B+/AA}(t-1)f_{BB/A+}(t-1)\Phi_F\\
&+\frac{1}{4}m_{BB/A+}(t-1)f_{B+/A+}(t-1)\Phi_F(1-\Phi_M)+\frac{1}{2}m_{BB/AA}(t-1)f_{B+/A+}(t-1)\Phi_F\\
&+\frac{1}{4}m_{B+/A+}(t-1)f_{++/A+}(t-1)\Phi_M+\frac{1}{2}m_{B+/A+}(t-1)f_{++/AA}(t-1)\Phi_M\\
&+\frac{1}{4}m_{B+/A+}(t-1)f_{B+/A+}(t-1)\Phi_M(1-\Phi_F)+\frac{1}{2}m_{B+/A+}(t-1)f_{B+/AA}(t-1)\Phi_M\\
&+\frac{1}{4}m_{B+/A+}(t-1)f_{BB/A+}(t-1)\Phi_M(1-\Phi_F)+\frac{1}{2}m_{B+/A+}(t-1)f_{BB/AA}(t-1)\Phi_M\\
&+\frac{1}{2}m_{BB/A+}(t-1)f_{++/A+}(t-1)\Phi_M+m_{BB/A+}(t-1)f_{++/AA}(t-1)\Phi_M\\
&+\frac{1}{4}m_{BB/A+}(t-1)f_{B+/A+}(t-1)\Phi_M(1-\Phi_F)+\frac{1}{2}m_{BB/A+}(t-1)f_{B+/AA}(t-1)\Phi_M\\
&+\frac{1}{2}m_{B+/A+}(t-1)f_{B+/A+}(t-1)\Phi_M\Phi_F+\frac{1}{2}m_{B+/A+}(t-1)f_{BB/A+}(t-1)\Phi_M\Phi_F\\
&+\left.\frac{1}{2}m_{BB/A+}(t-1)f_{B+/A+}(t-1)\Phi_M\Phi_F\right]/\tilde{\Omega},
\end{aligned}
$$

$$
\begin{aligned}
m_{BB/++}=\frac{\Omega_{BB/++}}{2}&\left[\frac{1}{4}m_{B+/++}(t-1)f_{B+/++}(t-1)+\frac{1}{8}m_{B+/++}(t-1)f_{B+/A+}(t-1)(1-\Phi_F)\right. \quad (13)\\
&+\frac{1}{2}m_{B+/++}(t-1)f_{BB/++}(t-1)+\frac{1}{4}m_{B+/++}(t-1)f_{BB/A+}(t-1)(1-\Phi_F)\\
&+\frac{1}{8}m_{B+/A+}(t-1)(1-\Phi_M)f_{B+/++}(t-1)+\frac{1}{16}m_{B+/A+}(t-1)(1-\Phi_M)f_{B+/A+}(t-1)(1-\Phi_F)\\
&+\frac{1}{4}m_{B+/A+}(t-1)(1-\Phi_M)f_{BB/++}(t-1)+\frac{1}{8}m_{B+/A+}(t-1)(1-\Phi_M)f_{BB/A+}(t-1)(1-\Phi_F)\\
&+\frac{1}{2}m_{BB/++}(t-1)f_{B+/++}(t-1)+\frac{1}{4}m_{BB/++}(t-1)f_{B+/A+}(t-1)(1-\Phi_F)\\
&+m_{BB/++}(t-1)f_{BB/++}(t-1)+\frac{1}{2}m_{BB/++}(t-1)f_{BB/A+}(t-1)(1-\Phi_F)\\
&+\frac{1}{4}m_{BB/A+}(t-1)(1-\Phi_M)f_{B+/++}(t-1)+\frac{1}{8}m_{BB/A+}(t-1)(1-\Phi_M)f_{B+/A+}(t-1)(1-\Phi_F)\\
&+\left.\frac{1}{2}m_{BB/A+}(t-1)(1-\Phi_M)f_{BB/++}(t-1)+\frac{1}{4}m_{BB/A+}(t-1)(1-\Phi_M)f_{BB/A+}(t-1)(1-\Phi_F)\right]/\tilde{\Omega}
\end{aligned}
$$

$$
\begin{aligned}
m_{BB/A+} = \frac{\Omega_{BB/A+}}{2} \Bigg[& \frac{1}{8} m_{B+/++}(t-1) f_{B+/A+}(t-1)(1-\Phi_F) + \frac{1}{4} m_{B+/++}(t-1) f_{B+/AA}(t-1) \\
& + \frac{1}{4} m_{B+/++}(t-1) f_{BB/A+}(t-1)(1-\Phi_F) + \frac{1}{2} m_{B+/++}(t-1) f_{BB/AA}(t-1) \\
& + \frac{1}{8} m_{B+/A+}(t-1)(1-\Phi_M) f_{B+/++}(t-1) + \frac{1}{8} m_{B+/A+}(t-1)(1-\Phi_M) f_{B+/A+}(t-1)(1-\Phi_F) \\
& + \frac{1}{8} m_{B+/A+}(t-1)(1-\Phi_M) f_{B+/AA}(t-1) + \frac{1}{4} m_{B+/A+}(t-1)(1-\Phi_M) f_{BB/++}(t-1) \\
& + \frac{1}{4} m_{B+/A+}(t-1)(1-\Phi_M) f_{BB/A+}(t-1)(1-\Phi_F) + \frac{1}{4} m_{B+/A+}(t-1)(1-\Phi_M) f_{BB/AA}(t-1) \\
& + \frac{1}{4} m_{B+/AA}(t-1) f_{B+/++}(t-1) + \frac{1}{8} m_{B+/AA}(t-1) f_{B+/A+}(t-1)(1-\Phi_F) \\
& + \frac{1}{2} m_{B+/AA}(t-1) f_{BB/++}(t-1) + \frac{1}{4} m_{B+/AA}(t-1) f_{BB/A+}(t-1)(1-\Phi_F) \\
& + \frac{1}{4} m_{BB/++}(t-1) f_{B+/A+}(t-1)(1-\Phi_F) + \frac{1}{2} m_{BB/++}(t-1) f_{B+/AA}(t-1) \\
& + \frac{1}{2} m_{BB/++}(t-1) f_{BB/A+}(t-1)(1-\Phi_F) + m_{BB/++}(t-1) f_{BB/AA}(t-1) \\
& + \frac{1}{4} m_{BB/A+}(t-1)(1-\Phi_M) f_{B+/++}(t-1) + \frac{1}{4} m_{BB/A+}(t-1)(1-\Phi_M) f_{B+/A+}(t-1)(1-\Phi_F) \\
& + \frac{1}{4} m_{BB/A+}(t-1)(1-\Phi_M) f_{B+/AA}(t-1) + \frac{1}{2} m_{BB/A+}(t-1)(1-\Phi_M) f_{BB/++}(t-1) \\
& + \frac{1}{2} m_{BB/A+}(t-1)(1-\Phi_M) f_{BB/A+}(t-1)(1-\Phi_F) + \frac{1}{2} m_{BB/A+}(t-1)(1-\Phi_M) f_{BB/AA}(t-1) \\
& + \frac{1}{2} m_{BB/AA}(t-1) f_{B+/++}(t-1) + \frac{1}{4} m_{BB/AA}(t-1) f_{B+/A+}(t-1)(1-\Phi_F) \\
& + m_{BB/AA}(t-1) f_{BB/++}(t-1) + \frac{1}{2} m_{BB/AA}(t-1) f_{BB/A+}(t-1)(1-\Phi_F) \\
& + \frac{1}{4} m_{B+/++}(t-1) f_{B+/A+}(t-1)\Phi_F + \frac{1}{2} m_{B+/++}(t-1) f_{BB/A+}(t-1)\Phi_F \\
& + \frac{1}{8} m_{B+/A+}(t-1) f_{B+/A+}(t-1)\Phi_F(1-\Phi_M) + \frac{1}{8} m_{B+/A+}(t-1) f_{BB/A+}(t-1)\Phi_F(1-\Phi_M) \\
& + \frac{1}{2} m_{BB/++}(t-1) f_{B+/A+}(t-1)\Phi_F + m_{BB/++}(t-1) f_{BB/A+}(t-1)\Phi_F \\
& + \frac{1}{4} m_{BB/A+}(t-1) f_{B+/A+}(t-1)\Phi_F(1-\Phi_M) + \frac{1}{2} m_{BB/A+}(t-1) f_{BB/A+}(t-1)\Phi_F(1-\Phi_M) \\
& + \frac{1}{4} m_{B+/A+}(t-1) f_{B+/++}(t-1)\Phi_M + \frac{1}{8} m_{B+/A+}(t-1) f_{B+/A+}(t-1)\Phi_M(1-\Phi_F) \\
& + \frac{1}{2} m_{B+/A+}(t-1) f_{BB/++}(t-1)\Phi_M + \frac{1}{4} m_{B+/A+}(t-1) f_{BB/A+}(t-1)\Phi_M(1-\Phi_F) \\
& + \frac{1}{2} m_{BB/A+}(t-1) f_{B+/++}(t-1)\Phi_M + \frac{1}{4} m_{BB/A+}(t-1) f_{B+/A+}(t-1)\Phi_M(1-\Phi_F) \\
& + m_{BB/A+}(t-1) f_{BB/++}(t-1)\Phi_M + \frac{1}{2} m_{BB/A+}(t-1) f_{BB/A+}(t-1)\Phi_M(1-\Phi_F) \Bigg] / \tilde{\Omega},
\end{aligned} \tag{14}
$$

$$
\begin{aligned}
m_{BB/AA} = \frac{\Omega_{BB/AA}}{2} \Bigg[& \frac{1}{16} m_{B+/A+}(t-1)(1-\Phi_M) f_{B+/A+}(t-1)(1-\Phi_F) \\
& + \frac{1}{8} m_{B+/A+}(t-1)(1-\Phi_M) f_{B+/AA}(t-1) + \frac{1}{8} m_{B+/A+}(t-1)(1-\Phi_M) f_{BB/A+}(t-1)(1-\Phi_F) \\
& + \frac{1}{4} m_{B+/A+}(t-1)(1-\Phi_M) f_{BB/AA}(t-1) + \frac{1}{8} m_{B+/AA}(t-1) f_{B+/A+}(t-1)(1-\Phi_F) \\
& + \frac{1}{4} m_{B+/AA}(t-1) f_{B+/AA}(t-1) + \frac{1}{4} m_{B+/AA}(t-1) f_{BB/A+}(t-1)(1-\Phi_F) \\
& + \frac{1}{2} m_{B+/AA}(t-1) f_{BB/AA}(t-1) + \frac{1}{8} m_{BB/A+}(t-1)(1-\Phi_M) f_{B+/A+}(t-1)(1-\Phi_F) \\
& + \frac{1}{4} m_{BB/A+}(t-1)(1-\Phi_M) f_{B+/AA}(t-1) + \frac{1}{4} m_{BB/A+}(t-1)(1-\Phi_M) f_{BB/A+}(t-1)(1-\Phi_F)
\end{aligned} \tag{15}
$$

$$+\frac{1}{2}m_{BB/A+}(t-1)(1-\Phi_M)f_{BB/AA}(t-1)+\frac{1}{4}m_{BB/AA}(t-1)f_{B+/A+}(t-1)(1-\Phi_F)$$
$$+\frac{1}{2}m_{BB/AA}(t-1)f_{B+/AA}(t-1)+\frac{1}{2}m_{BB/AA}(t-1)f_{BB/A+}(t-1)(1-\Phi_F)$$
$$+m_{BB/AA}(t-1)f_{BB/AA}(t-1)+\frac{1}{8}m_{B+/A+}(t-1)f_{B+/A+}(t-1)\Phi_F(1-\Phi_M)$$
$$+\frac{1}{8}m_{B+/A+}(t-1)f_{BB/A+}(t-1)\Phi_F(1-\Phi_M)+\frac{1}{4}m_{B+/AA}(t-1)f_{B+/A+}(t-1)\Phi_F$$
$$+\frac{1}{2}m_{B+/AA}(t-1)f_{BB/A+}(t-1)\Phi_F+\frac{1}{4}m_{BB/A+}(t-1)f_{B+/A+}(t-1)\Phi_F(1-\Phi_M)$$
$$+\frac{1}{2}m_{BB/A+}(t-1)f_{BB/A+}(t-1)\Phi_F(1-\Phi_M)+\frac{1}{2}m_{BB/AA}(t-1)f_{B+/A+}(t-1)\Phi_F$$
$$+m_{BB/AA}(t-1)f_{BB/A+}(t-1)\Phi_F+\frac{1}{8}m_{B+/A+}(t-1)f_{B+/A+}(t-1)\Phi_M(1-\Phi_F)$$
$$+\frac{1}{4}m_{B+/A+}(t-1)f_{B+/AA}(t-1)\Phi_M+\frac{1}{4}m_{B+/A+}(t-1)f_{BB/A+}(t-1)\Phi_M(1-\Phi_F)$$
$$+\frac{1}{2}m_{B+/A+}(t-1)f_{BB/AA}(t-1)\Phi_M+\frac{1}{4}m_{BB/A+}(t-1)f_{B+/A+}(t-1)\Phi_M(1-\Phi_F)$$
$$+\frac{1}{2}m_{BB/A+}(t-1)f_{B+/AA}(t-1)\Phi_M+\frac{1}{2}m_{BB/A+}(t-1)f_{BB/A+}(t-1)\Phi_M(1-\Phi_F)$$
$$+m_{BB/A+}(t-1)f_{BB/AA}(t-1)\Phi_M+\frac{1}{4}m_{B+/A+}(t-1)f_{B+/A+}(t-1)\Phi_M\Phi_F$$
$$+\frac{1}{2}m_{B+/A+}(t-1)f_{BB/A+}(t-1)\Phi_M\Phi_F+\frac{1}{2}m_{BB/A+}(t-1)f_{B+/A+}(t-1)\Phi_M\Phi_F$$
$$+m_{BB/A+}(t-1)f_{BB/A+}(t-1)\Phi_M\Phi_F\big]/\tilde{\Omega},$$

and nine identical equations for females of these genotypes. Here *m* and *f* represent males and females of the genotype indicated in their respective subscripts and Ω is the fitness of a given genotype (in subscript) relative to wild-type. The overall fitness of the entire population Ω is calculated as the sum of˜ all numerators in equations (7)-(15) and is used as a normalising factor to ensure genotype frequencies fill the entire range from zero to one.

To predict the results of the split drive treatment cages (main text) we compare the sum of squared errors for B, A and B/A carriers to model results for additive or multiplicative combinations of the relative fitness parameters obtained in Section 1. These produced very similar goodness of fit and so an additive combination of fitness parameters was utilized since this yields a simple and intuitive explanation of fitness cost interactions. The particular model scenario considered is that in which the initial cage setup consists of females heterozygous for both transgenic constructs and wild-type males, leading to initial conditions of the form

$m_{++/++}(0) = 0.5$, $m_{++/A+}(0) = 0$, $m_{++/AA}(0) = 0$, $m_{B+/++}(0) = 0$, $m_{B+/A+}(0) = 0$,

$m_{B+/AA}(0) = 0$, $m_{BB/++}(0) = 0$, $m_{BB/A+}(0) = 0$, $m_{BB/AA}(0) = 0$, $f_{++/++}(0) = 0$,

$f_{++/A+}(0) = 0$, $f_{++/AA}(0) = 0$, $f_{B+/++}(0) = 0$, $f_{B+/A+}(0) = 0.5$, $f_{B+/AA}(0) = 0$,

$f_{BB/++}(0) = 0$, $f_{BB/A+}(0) = 0$, $f_{BB/AA}(0) = 0$.

All numerical simulations of this model were performed using Matlab (version R2019a; The MathWorks Inc., Natick, MA).

2.5 Split-Drive Stochastic Model

The deterministic model outlined above was used to (1) estimate relative fitness parameters for each transgenic construct and (2) to provide simple, initial predictions around the outcome of the split drive treatment cages. While useful in planning cage trials and to some extent in analyzing the final outcomes, such models do not account for the stochasticity inherent in such experiments. Thus, we also developed a stochastic model framework capable of providing an estimated range within which we would expect the experimental results to fall. This is largely based on a previously developed model framework used for the study of novel multiplexing approaches for CRISPR-based gene drives[1] and has been altered to capture the dynamics of a split drive system. The main basis of this model has been discussed in depth previously and so here we provide only a brief outline of the adjustments made.

Firstly, since the initial model was formulated for the study of multiplexing strategies, it was already capable of managing genetic inheritance across multiple loci. Rather than allowing a free choice of number of loci, here we eliminate this and fix the model to two loci (those for the A and B elements of the split drive).

Perhaps the most important alteration here is the switch from the initial model of embryonic homing to one considering germline homing - as observed in the split drive developed in this study. In the initial model, a homing module altered the assigned genotypes of 'eggs' resulting from each mating pair. To move to a model of germline homing, this homing module was simply moved such that it acts between the selection of parental mating pairs and the assignment of genotypes to the resulting 'eggs' from those mating pairs.

The final, and rather trivial, change required here was in the calculation of transgene carrier frequencies. In the initial model these were calculated in the context of multiplexing approaches where the presence of any gene drive construct was the important factor - thus resulting in a single transgene carrier frequency no matter the multiplex number. However, here it is important to track carrier frequencies of both the B and A elements of the split drive. The relevant calculations were adjusted accordingly, producing separate transgene carrier frequencies for the B and A elements.

As with the deterministic modelling, all stochastic numerical simulations were performed using Matlab (version R2019a; The MathWorks Inc., Natick, MA).

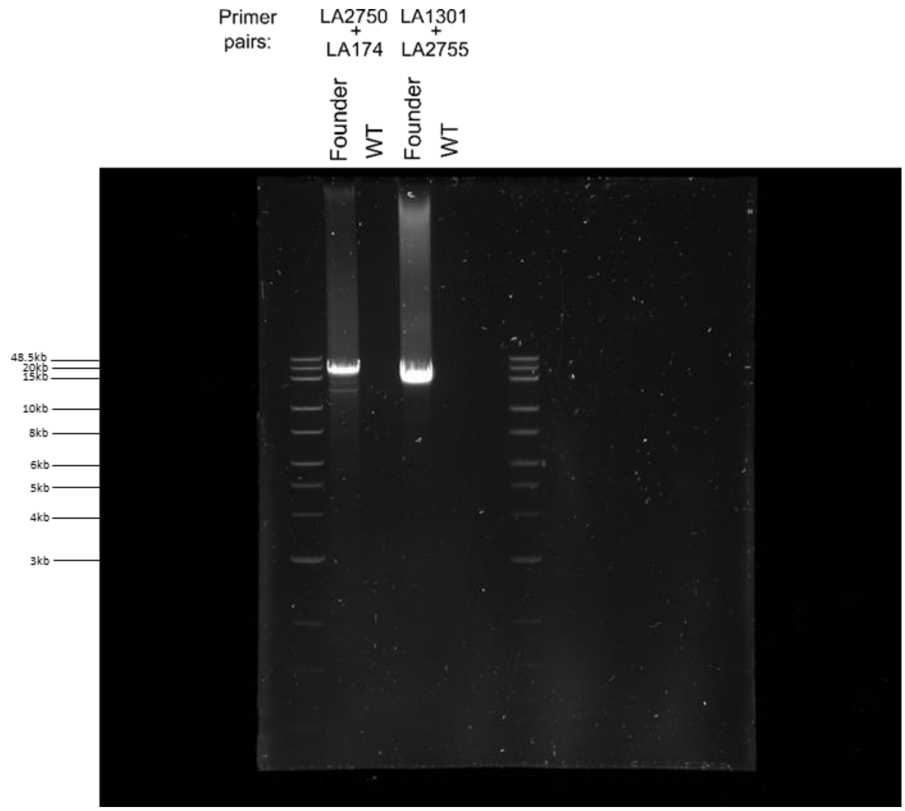

Fig S4. Confirmation of *kmo*^{sgRNAs} integration site by PCR. Gel electrophoresis of PCRs to confirm the integration site of *kmo*^{sgRNAs}. The Quick Load 1kb Extend DNA ladder (NEB) was loaded into the first and last well. Expected amplicons are 10,897bp and 16,306bp.

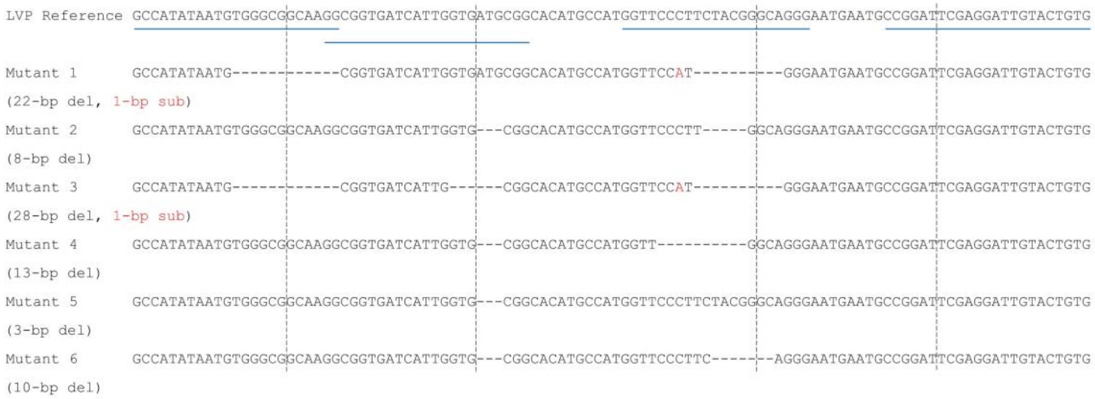

Fig S5. Mutations identified in the kmo$^{-/-}$ line. CRISPResso2 analysis of four pooled samples of 5 individuals from the kmo$^{-/-}$ line revealed six different mutations present in the line.

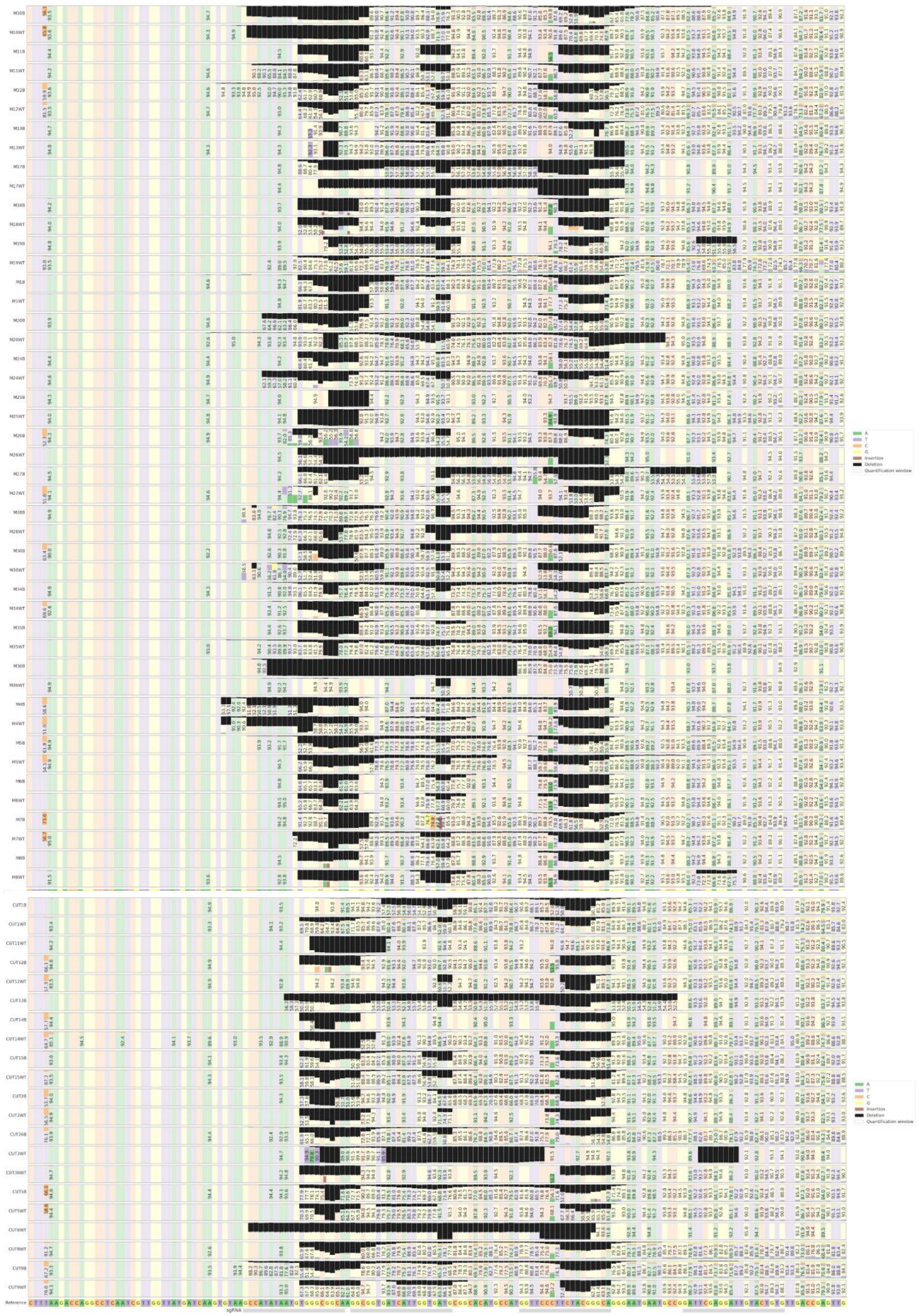

Figure S6. CRISPRESSO analysis reveals distinct cuts at each target site within individuals.

Alignment profiles for samples presented in Fig 2 of main text. Top section presents the white eyed F_2 progeny of female trans-heterozygotes which did not inherit the *kmo*^{sgRNAs}. Bottom section presents the white eyed F_2 progeny of male trans-heterozygotes which did not inherit the *kmo*^{sgRNAs}. Each line presents the sequencing results from a single individual and the black bar and number in each position represents the proportion of reads which were non-WT at that nucleotide for that sample. As these individuals have two kmo alleles, a fully black bar means that both alleles are mutated at that position, approximately 50% indicates one allele was mutant at that position and any other values indicate mosaicism in that individual. sgRNA targets (not including the PAM) are indicated by grey bars under the reference sequence.

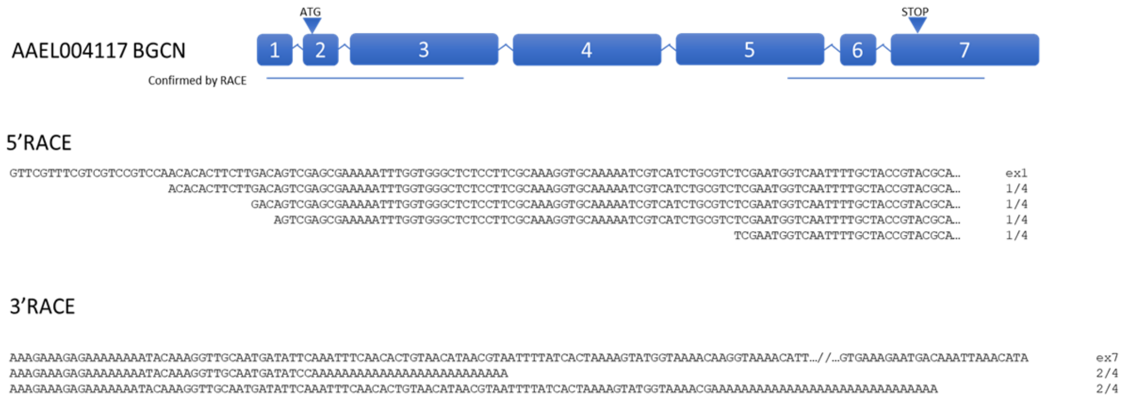

Fig S7. RACE results for *bgcn*. Diagram of the *bgcn* gene based on Vectorbase L5 assembly annotations (not to scale). RACE PCR products were cloned into the pJET vector and four clones were sequenced. The top line denotes the start of exon 1 or end of exon 7 as annotated, proceeding lines begin with the first base transcribed (pJET vector and RACE primer sequences trimmed). Right hand column denotes the number of clones with identical sequences.

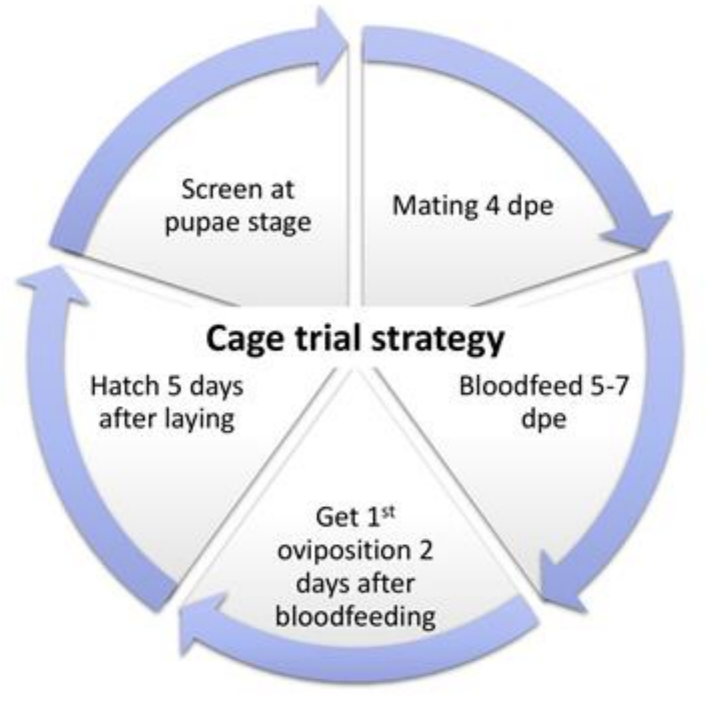

Fig S8. Diagram of the cage trial strategy. Steps followed during the 6 generations of the cage trial beginning with the mating of males and females 4 days post eclosion (dpe). Females were allowed to blood feed when they were 5-7 dpe and 2 days later eggs were collected. Five days after oviposition the eggs were hatched in degassed RO (reverse osmosis) water. 250 larvae for each cage were reared under standardized conditions. Finally, the progeny of each generation was screened at pupae stage for fluorescence and eye phenotype.

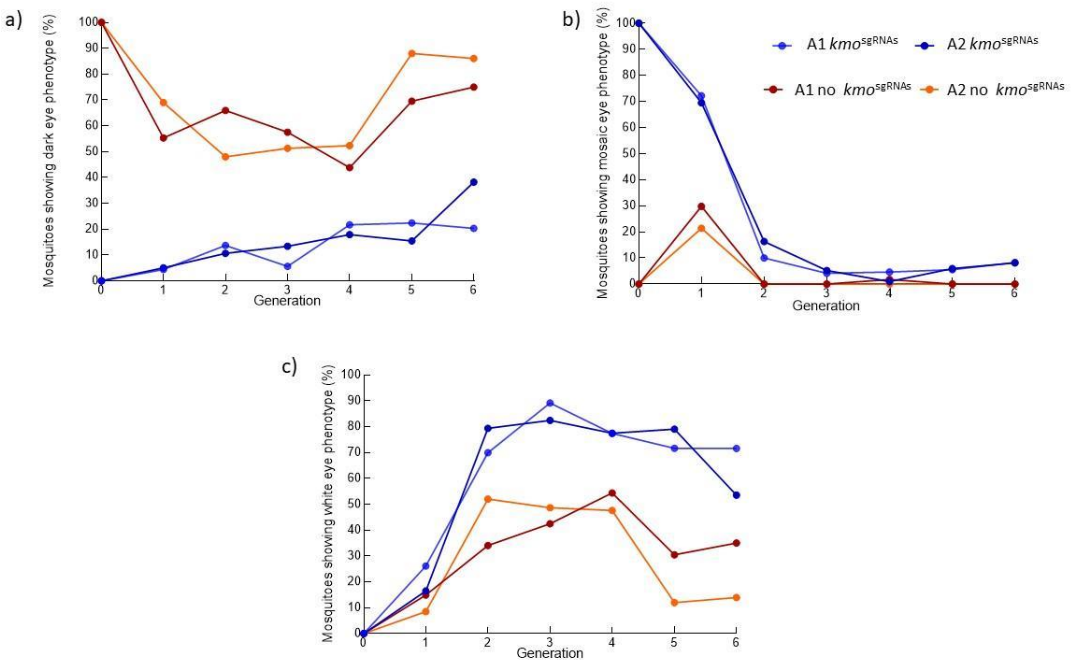

Figure S9. Eye phenotypes tracked through cage trial. Proportion of mosquitoes presenting dark eyes (a), mosaic eyes (b) or white eyes (c) screened from each generation of the cage trial experimental cages A1 and A2. Phenotypes have been separated based on the inheritance of the *kmo*^{sgRNAs} element. Blue lines indicate those which inherited the *kmo*^{sgRNAs} element; these would require disruption of one *kmo* allele to appear as mosaic (somatic disruption) or white eyed (germline disruption inherited from parents). Those which do not carry the *kmo*^{sgRNAs} element are indicated in orange and can be either completely non-transgenic or carry the *bgcn*-Cas9. In these individuals two alleles of *kmo* would need to be disrupted to present a mosaic or white eyed phenotype.

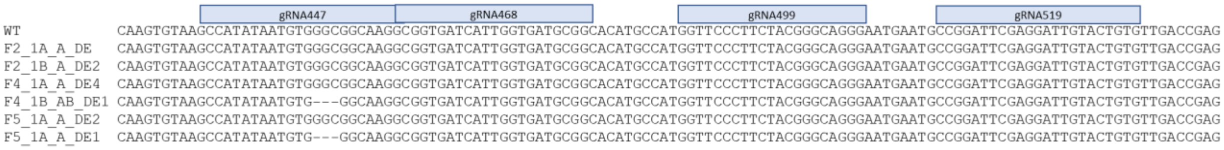

Fig S10. Mutational profiles of dark-eyed individuals identified in later generations of the cage trial. Dark eyed samples which also have the kmo^{sgRNAs} transgene were collected from the cage trial and Sanger sequenced. FX – generation of cage trial, 1Y – replicate experimental cage (A or B), DE - dark eyes, A - kmo^{sgRNAs}, AB - kmo^{sgRNAs};*bgcn*-Cas9.

Table S1. Summary of microinjections performed to establish transgenic lines.

Construct	Embryos injected	G_0 survivors (%)	G_1s screened	Positive G_1s
kmo^{sgRNAs} (AGG1095)	1972	60 (3%)	6345	46 (3/4 pools)
bgcn-Cas9 (AGG1207)	1152	129 (11.2%)	2238	38 (5/6 pools)

Table S2. **Homozygous viability of *bgcn*-Cas9 line.**

Cross	No. progeny inheriting *bgcn*-Cas9 (%)	No. WT progeny	TOTAL screened	Chi-square value	P value*
bgcn-Cas9	202 (74%)	69	271	0.0196	0.8886

WT: wild type

*Chi-square test (df.1, p<0.0001) for deviation from expected Mendelian inheritance rate.

Table S3. F_1 progeny from the *bgcn*-Cas9 x *kmo*^{sgRNAs} F_0 crosses.

F_0 Cross (female x male)	No. F_1 positive for *kmo*^{sgRNAs} (mosaics)	No. F_1 positive for *kmo*^{sgRNAs}; *bgcn*-Cas9 (mosaics)	No. F_1 positive for *bgcn*-Cas9 (mosaics)	No. WT F_1 (mosaics)	Total F1 scored
bgcn-Cas9D x *kmo*^{sgRNAs}	29 (17)	34 (27)	23 (0)	29 (0)	115 (44)

WT: wild type

Table S4. Inheritance assessments of progeny from *kmo*^{sgRNAs}; *bgcn*-Cas9 transheterozygous F_1.

F_1 Cross (female x male)	No. F_2 positive for *kmo*^{sgRNAs}	No. F_2 positive for *kmo*^{sgRNAs}; *bgcn*-Cas9	No. F_2 positive for *bgcn*-Cas9	No. WT F_2	Total F_2 scored	Total F_2 inheriting *kmo*^{sgRNAs} (%)	Total F_2 inheriting *bgcn*-Cas9 (%)
WT x *kmo*^{sgRNAs}; *bgcn*-Cas9D (exp 1)	33	44	20	16	113	77 (68.1)	64 (56.6)
WT x *kmo*^{sgRNAs}; *bgcn*-Cas9D (exp 2)	431	367	155	618	1571	798 (50.8)	522 (33.2)
WT x *kmo*^{sgRNAs}; *bgcn*-Cas9D (exp 3)	213	198	154	190	755	411 (54.4)	352 (46.7)
kmo^{sgRNAs}; *bgcn*-Cas9D x WT (exp 1)	122	123	47	26	318	245 (77)	170 (53.45
kmo^{sgRNAs}; *bgcn*-Cas9D x WT (exp 2)	66	45	17	19	147	111 (75.5)	62 (42.2)
kmo^{sgRNAs}; *bgcn*-Cas9D x WT (exp 3)	157	162	43	46	408	319 (78.7)	205 (50.2)

WT: wild type

Table S5. Mosaicism assessments of progeny from *kmo*^{sgRNAs}; *bgcn*-Cas9 trans-heterozygous F_1.

F_1 Cross (female x male)	No. mosaics in the F_2 positive for *kmo*^{sgRNAs} (%)	No. mosaics in the F_2 positive for *kmo*^{sgRNAs}; *bgcn*-Cas9 (%)	No. mosaics in the F_2 positive for *bgcn*-Cas9 (%)	No. mosaics in the WT F_2 (%)
WT x *kmo*^{sgRNAs}; *bgcn*-Cas9D (exp 1)	2 (6)	20 (45.5)	0	0
WT x *kmo*^{sgRNAs}; *bgcn*-Cas9D (exp 2)	104 (21.1)	235 (64)	0	0
WT x *kmo*^{sgRNAs}; *bgcn*-Cas9D (exp 3)	25 (11.7)	56 (28.3)	0	0
kmo^{sgRNAs}; *bgcn*-Cas9D x WT (exp 1)	76 (62.3)	87 (71)	9 (19.1)	1 (3.8)
kmo^{sgRNAs}; *bgcn*-Cas9D x WT (exp 2)	65 (98.5)	45 (100)	3 (17.6)	3 (15.8)
kmo^{sgRNAs}; *bgcn*-Cas9D x WT (exp 3)	148 (94.3)	157 (97)	19 (44.2)	15 (32.6)

WT: wild type

Table S6. G-test result data for F_2 rates of inheritance. This is a maximum likelihood significance test. Individual crosses are analyzed for significant deviation from Mendelian inheritance. Individual crosses are pooled according to treatment for an overall Goodness of fit G-value and scored for whether there is heterogeneity among treatments.

Line	df	G – goodness of fit	P-value
Male - Mosaics	1	15.221	0.000096*
Female - Mosaics	1	98.201	3.78E-23*

Pooled crosses	df	G – goodness of fit	P-value	df	Heterogeneity G-value	P-value
D Line	1	110.03	2.2E-16	2	3.39	0.066

Table S7. Model summaries for estimates of homing and cutting rates from *kmo*^{sgRNAs}; *bgcn* Cas9 mosquitoes crossed to WT mosquitoes. Odds coefficients, and 95% confidence intervals, and significance values taken from a binomial glm with 'logit' link. Two models compare the calculated odds of observing *kmo*^{sgRNAs} inheritance and white eyes.

	kmo^{sgRNAs} inheritance			white eyes		
Predictors	*Odds*	*95% CI*	*p*	*Odds*	*95% CI*	*p*
(Intercept)	4.31	3.66 – 5.09	<0.001	4.90	4.14 – 5.80	<0.001
N		59			53	

Table S8. Summary of the cutting assay screening from *kmo*^{sgRNAs};*bgcn* Cas9 mosquitoes crossed to *kmo*$^{-/-}$ mosquitoes.

Cross (female x male)	Replicate	No. F_2 positive for *kmo*^{sgRNAs}	No. F_2 positive for *kmo*^{sgRNAs}; *bgcn*-Cas9	No. F_2 positive for *bgcn*-Cas9	No. WT F_2	Total F_2 scored	Total F_2 inheriting *kmo*^{sgRNAs}	Total F_2 non-inheriting *kmo*^{sgRNAs}	Total F_2 non-inheriting *kmo*^{sgRNAs} with WE
(*kmo*^{sgRNAs}; *bcgn*-Cas9) x kmo$^{-/-}$	1	10	17	4	4	35	27	8	8
	2	16	6	8	12	42	22	20	20
	3	12	7	14	16	49	19	30	30
	4	23	22	13	8	66	45	21	5
	5	9	11	1	7	28	20	8	8
	6	22	35	6	6	69	57	12	12
	7	6	16	10	3	35	22	13	13
	8	26	24	17	10	77	50	27	13
	9	13	30	14	21	78	43	35	30
	10	33	38	4	1	76	71	5	5
	12	25	24	3	1	53	49	4	4
	13	43	25	12	11	91	68	23	20
	14	22	20	2	3	47	42	5	4
	16	16	19	1	0	36	35	1	0
	17	21	18	8	14	61	39	22	22
	18	4	10	1	4	19	14	5	5
	19	8	6	2	2	18	14	4	4
	20	16	14	2	7	39	30	9	9
	21	12	12	1	3	28	24	4	4

	22	5	7	3	9	24	12	12	12
	23	21	13	8	6	48	34	14	12
	24	4	7	10	3	24	11	13	9
	25	31	35	4	7	77	66	11	6
	26	9	4	1	1	15	13	2	2
kmo$^{-/-}$ x (*kmo*$^{sgRNAs;}$ *bcgn*-Cas9)	1	19	18	14	26	77	37	40	9
	2	14	7	10	6	37	21	16	15
	3	56	63	1	2	122	119	3	0
	4	10	17	17	8	52	27	25	15
	5	19	10	8	12	49	29	20	1
	6	27	20	22	20	89	47	42	7
	7	27	20	1	1	49	47	2	2
	8	16	22	18	20	76	38	38	6
	9	44	50	0	2	96	94	2	2
	10	19	27	18	9	73	46	27	7
	11	27	30	9	11	77	57	20	14
	12	30	26	23	29	108	56	52	2
	13	24	29	10	9	72	53	19	19
	14	19	11	18	15	63	40	33	8
	15	11	15	13	13	52	26	26	0
	16	2	3	1	3	9	5	4	1
	17	7	4	3	2	16	11	5	4

	18	2	5	5	2	14	7	7	1
	19	34	30	0	1	65	64	1	1
	20	50	33	0	0	83	83	0	0
	21	20	13	0	0	33	33	0	0
	22	16	14	6	10	46	30	16	16

WT: wild type; WE: white-eyed

Table S9. Full model summaries for estimates of homing and cutting rates from *kmo*^{sgRNAs}; *bgcn* Cas9 mosquitoes crossed to *kmo*$^{-/-}$ mosquitoes. Log-odds and 95% confidence intervals, and significance values are taken from a binomial glm or glmm with 'logit' link using sex of the parent as a fixed effect. Mixed-effects models included replicate as a nested random factor within the sex of the parent.

	1.Mixed-effects model Homing rates	2.Mixed-effects model Combined male & female homing rates	3.Mixed-effects model Cutting rates	4.Pooled model for Homing rates	5.Pooled model for Cutting rates
Predictors	*Log-Odds*	*Log-Odds*	*Log-Odds*	*Log-Odds*	*Log-Odds*
(Intercept)	1.22 *** (0.70 – 1.74)	1.18 *** (0.82 – 1.53)	2.65 *** (1.57 – 3.72)	0.92 *** (0.80 – 1.04)	1.45 *** (1.32 – 1.59)
kmo^{sgRNAs}; *bcgn*-Cas9♀ x *kmo*$^{-/-}$ ♂	-0.09 (-0.80 – 0.63)	N/A	2.35 ** (0.77 – 3.93)	0.07 (-0.10 – 0.25)	1.61 *** (1.30 – 1.93)
Random Effects					
σ^2	4.64	4.64	8.38		
τ_{00}	1.35 Replicate:Cross	1.35 Replicate:Cross	5.09 Replicate:Cross		
	0.00 Cross	0.00 Cross	0.00 Cross		
N	26 Replicate	26 Replicate	26 Replicate		
	2 Cross	2 Cross	2 Cross		
Observations	46	46	46	46	46
Marginal R^2 / Conditional R^2	0.000 / NA	0.000/ NA	0.144 / NA	0.007	0.073

* p<0.05 ** p<0.01 *** p<0.001

Table S10. Number of reads for each sample analysed by CRISPResso2.

Sample name	READS IN INPUTS	READS AFTER PREPROCESSING	READS ALIGNED
LVP1	15463	2630	2630
LVP2	12663	2751	2751
LVP3	19848	4501	4500
kmoKO5m	199913	52857	52857
kmoKO8m	23977	5316	5316
kmoKO5f	173303	37919	37919
kmoKO6f	16144	3932	3932
F2_1A_AB_ME1	74605	13875	13646
F2_1A_AB_ME2	37813	6430	6277
F2_1A_AB_WE1	47942	6769	6160
F2_1A_AB_WE2	27971	4134	3720
F2_1A_A_WE	39313	7238	7234
F2_1A_B_WE	49228	9175	9175
F2_1A_WT_WE1	46068	10926	10926
F2_1A_WT_WE2	46004	9150	9150
F2_1B_AB_ME1	9424	1749	1666
F2_1B_AB_ME2	42933	7155	6875
F2_1B_AB_ME3	37963	4410	4313
F2_1B_A_WE1	4608	747	747
F2_1B_WT_WE1	9133	1584	1584
F4_1A_AB_ME	44162	7903	7735
F4_1A_AB_WE*	58	2	2
F4_1A_A_WE	2666	621	621
F4_1A_B_WE1	7083	1228	1227
F4_1A_B_WE2	149540	34140	34140
F4_1A_B_WE3	63642	14201	14201
F4_1A_WT_WE1	62630	6359	6359
F4_1A_WT_WE2	12980	2176	2176
F4_1B_AB_WE*	172	9	9
F4_1B_A_WE	47872	8480	8480
F5_1A_AB_ME1	1171441	193255	190179
F5_1A_AB_ME2	35357	7009	6884
F5_1A_AB_ME3	51823	11005	11005
F5_1A_AB_ME4	31550	1230	1221
F5_1A_AB_ME5	27683	5556	5457
F5_1A_AB_ME6	38129	5974	5806
F5_1A_AB_WE1	41588	3307	3228
F5_1A_AB_WE2	24837	7881	7832
F5_1A_AB_WE3	47028	7907	7907
F5_1A_AB_WE4	40179	7986	7332

F5_1A_AB_WE5	43165	5759	5759
F5_1A_A_ME1	29189	6404	6385
F5_1A_A_WE1	52947	12810	12810
F5_1A_A_WE2	27834	6719	6719
F5_1A_A_WE4	30055	5212	5212
F5_1A_A_WE5	23860	6650	6650
F5_1A_B_WE	54126	9830	9537
F5_1A_WT_WE1	64447	13065	12523
F5_1A_WT_WE2	24712	6770	6770
F5_1A_WT_WE3	55185	13751	13751
F5_1A_WT_WE4	34089	9304	9304
F5_1A_WT_WE5	25393	6001	6001
F5_1B_AB_WE1	523	31	30
F5_1B_AB_WE3	13449	2615	2567
F5_1B_A_WE2	28242	8018	8018
F5_1B_A_WE3*	4120	1	1
F5_1B_A_WE4*	406	3	3
F5_1B_A_WE7	21592	4889	4889
F2_1A_AB_DE2	13784	3102	3068
F2_1A_AB_DE3	29718	7571	7571
F2_1A_A_DE	45674	12556	12556
F2_1B_AB_DE	48354	10148	10148
F2_1B_A_DE1	10038	2449	2449
F2_1B_A_DE2	9314	1713	1713
F4_1A_AB_DE	49865	14173	14173
F4_1A_AB_DE1	9244	2540	2519
F4_1A_A_DE1	53557	15612	15612
F4_1A_A_DE2	42279	12024	12019
F4_1A_A_DE3	3609	984	984
F4_1A_A_DE4	4205	1009	1009
F4_1B_AB_DE1	48594	10070	10070
F4_1B_AB_DE2	16930	2266	2249
F4_1B_AB_DE3	11432	1860	1860
F4_1B_A_DE1	5616	1142	1142
F4_1B_A_DE2	7598	1240	1240
F4_1B_A_DE3	976398	192699	192698
F5_1A_AB_DE	25278	5872	5872
F5_1A_A_DE	37830	9026	9015
F5_1A_A_DE1	28492	8570	8570
F5_1A_A_DE2	55262	13619	13619
F5_1A_A_DE3	30359	7416	7371
F5_1A_A_DE4	40626	6961	6961

F5_1A_A_DE5	17880	3881	3877
F5_1B_AB_DE	84081	21346	21303
F5_1B_AB_DE1	23633	5324	5250
F5_1B_AB_DE2	50624	13251	13215
F5_1B_AB_DE3	19088	4849	4841
F5_1B_AB_DE4	37346	9261	9261
F5_1B_AB_DE5	16791	3683	3683
F5_1B_AB_DE6	12038	2988	2988
F5_1B_AB_DE7	19171	4166	4166
F5_1B_AB_DE8	55450	15794	15741
F5_1B_A_DE1	18303	3671	3671
F5_1B_A_DE2	57524	15808	15801
F5_1B_A_DE3	25455	6488	6488
F5_1B_A_DE4	52922	12427	12427
F5_1B_A_DE5	22987	6059	6059
F5_1B_A_DE6	31165	8567	8567
F5_1B_A_DE7	23657	4064	4064
CUT1B	311819	295309	287050
CUT1WT	9121	8115	7761
CUT11WT	172587	163497	161784
CUT12B	5564	5058	5016
CUT12WT	5323	4950	4931
CUT13B	4316	3818	2851
CUT14B	123063	114199	113639
CUT14WT	35387	29274	28901
CUT15B	6888	6089	5964
CUT15WT	2970	2467	1829
CUT2B	733952	676303	443104
CUT2WT	651202	607611	373922
CUT26B	1327	1093	1001
CUT3WT	190427	181546	171822
CUT36WT	8457	7931	7887
CUT5B	373302	350230	325060
CUT5WT	578866	544684	541175
CUT6WT	313782	297736	246368
CUT8WT	768	555	512
CUT9B	9769	8307	6847
CUT9WT	192625	179432	135865
M10B	38122	34545	32059
M10WT	227889	210591	205562

M11B	164421	153763	152588
M11WT	13643	12694	12108
M12B	324273	298727	295128
M12WT	12670	11204	10008
M13B	245844	229285	220796
M13WT	239608	224501	222916
M17B	221539	207650	194190
M17WT	196989	183764	168176
M18B	187661	174325	168967
M18WT	261646	244268	242167
M19B	76069	72520	71966
M19WT	657	557	526
M1B	351130	336510	328527
M1WT	358939	342271	340391
M20B	99314	94598	93290
M20WT	72898	68799	64933
M24B	30620	28204	27931
M24WT	15760	14957	14048
M25B	54573	50462	50061
M25WT	39665	35663	35174
M26B	75529	71769	70993
M26WT	62770	55381	51822
M27B	84453	80544	77440
M27WT	53079	44641	44033
M28B	5913	5381	5308
M28WT	6669	5558	5405
M30B	5122	4476	4341
M30WT	5331	4915	4739
M34B	3613	3166	2925
M34WT	3424	3087	2961
M35B	6035	5358	5178
M35WT	5530	5068	4610
M36B	195069	184515	166119
M36WT	226049	195760	193451
M4B	198059	187571	183076
M4WT	317077	300509	296339
M5B	18222	16773	16292
M5WT	383071	361326	355510
M6B	89201	83877	83267

M6WT	165649	155881	155132
M7B	58524	54452	52348
M7WT	296430	277980	270906
M8B	288665	272643	271207
M8WT	140912	126723	124851

*****these samples were excluded from further analyses due to low number of sequencing reads (<10)

Table S11. Complete primer list.

Name	Sequence (5'-3')
LA174	AGGATGTCGAAGGAGAAGGCCAGG
LA182	GGCGACTGAGATGTCCTAAATGCAC
LA184	CAGACCGATAAAACACATGCGTCA
LA186	CAGCGACGGATTCGCGCTATTTAG
LA187	GTGTAGCGTGAAGACGACAGAA
LA518	GCCTCCTGAATCCAAATATGCTTGC
LA924	GCACCGAATCGGTGCCTGCCTTCCGGCATGATAACGGACTTGCCTTATTCCAA CTTGTCGTGCTGTTCCCAGCACGACTCTGGAAC
LA925	GAAATTAATACGACTCACTATAGGGCCATATAATGTGGGCGGCAGTTCCAGAG TCGTGCTGG
LA926	GAAATTAATACGACTCACTATAGGCACAGTACAATCCTCGAATCGTTCCAGAGT CGTGCTGG
LA1074	CGGCCATTTACGATCGGTGGGTTTGG
LA1075	GCGTTGTTCTGCGGTCCCCTGTTTTTG
LA1076	CTGGCCAATGTTACTGTGGCCGGCG
LA1275	TTATGATGATCGCCCTGCCC
LA1301	TGGCCTTCTCCTTCGACATCCTGT
LA1352	GATTACGCCAAGCTTGATGGTTCCTCATGACCTGCGCCGC
LA1725	TTTTGCGGCCGCCAACGTTGGGGCGTCATAAG
LA1726	TTTTCTCGAGGTTGGAGCTGTTTTCGTT
LA1737	TTTTTTAATTAAATCTTGATACGTCTCTTCATCAAGC
LA1738	TTTTGCGATCGCCCTCGAGCTATGTTTAATTTGTCATTCTTTCACATTG
LA2750	TAAGTGTTTCGCAGACGGCTTCA
LA2755	ACGCATGTGGGAGAACGATA

Table S12. Closest potential off-targets as determined by CHOPCHOP, lowercase nucleotides indicate mismatches

Target	Sequence	Potential off targets
kmo447	GCCATATAATGTGGGCGGCAAGG	none
kmo468	GGCGGTGATCATTGGTGATGCGG	GGCcGTtgTCATTGGTGATGGGG<br>CCGgATCcaCAATGATCACCGCC<br>CCAgATCACCAATGATCAgtGCC<br>CCCCAcCcCCAATGATCACCcCC<br>GGCGGaGATCtTTGcTGATGTGG
kmo499	GGTTCCCTTCTACGGGCAGGG	GGTaCCCTTCTgaGGGCAGGG
kmo519	CACAGTACAATCCTCGAATCCGG	CACgccACAATCtTCGtATCGGG

References

1. M.P. Edgington, T. Harvey-Samuel, L. Alphey, Population-level multiplexing: A promising strategy to manage the evolution of resistance against gene drives targeting a neutral locus. *Evol. Appl.* **13**, 1939–1948 (2020).
