# Supplementary material for: A multiplexed, confinable CRISPR/Cas9 gene drive can propagate in caged Aedes aegypti populations
Source: Nat Commun. 2024 Jan 25;15:729. doi: 10.1038/s41467-024-44956-2 (PMC10810878; doi:10.1038/s41467-024-44956-2)
Supplement: Supplementary file 3 — Reporting Summary [file 41467_2024_44956_MOESM3_ESM.pdf]

nature portfolio

Corresponding author(s): Luke Alphey

Last updated by author(s): Dec 8, 2023

Reporting Summary

Nature Portfolio wishes to improve the reproducibility of the work that we publish. This form provides structure for consistency and transparency in reporting. For further information on Nature Portfolio policies, see our Editorial Policies and the Editorial Policy Checklist.

Statistics

For all statistical analyses, confirm that the following items are present in the figure legend, table legend, main text, or Methods section.

n/a	Confirmed	
[ ]	[x]	The exact sample size (*n*) for each experimental group/condition, given as a discrete number and unit of measurement
[ ]	[x]	A statement on whether measurements were taken from distinct samples or whether the same sample was measured repeatedly
[ ]	[x]	The statistical test(s) used AND whether they are one- or two-sided<br>*Only common tests should be described solely by name; describe more complex techniques in the Methods section.*
[ ]	[x]	A description of all covariates tested
[ ]	[x]	A description of any assumptions or corrections, such as tests of normality and adjustment for multiple comparisons
[ ]	[x]	A full description of the statistical parameters including central tendency (e.g. means) or other basic estimates (e.g. regression coefficient) AND variation (e.g. standard deviation) or associated estimates of uncertainty (e.g. confidence intervals)
[ ]	[x]	For null hypothesis testing, the test statistic (e.g. *F*, *t*, *r*) with confidence intervals, effect sizes, degrees of freedom and *P* value noted<br>*Give P values as exact values whenever suitable.*
[x]	[ ]	For Bayesian analysis, information on the choice of priors and Markov chain Monte Carlo settings
[ ]	[x]	For hierarchical and complex designs, identification of the appropriate level for tests and full reporting of outcomes
[x]	[ ]	Estimates of effect sizes (e.g. Cohen's *d*, Pearson's *r*), indicating how they were calculated

Our web collection on statistics for biologists contains articles on many of the points above.

Software and code

Policy information about availability of computer code

Data collection

Complete details of the model available in Supplementary information S2. All Matlab scripts used in the mathematical modeling are freely available via the Open Science Framework (osf.io/bp4yh).

Data analysis

Amplicon-Seq analysis
Sequencing reads generated from the amplicon sequencing were analysed using the CRISPRessoBatch tool in CRISPResso2 (610 with the following script:

```
CRISPRessoBatch --batch_settings [batch file name] -a
ttatgatgatcgccctgcccaatcaggatcgcacttggacggtgacgctgttcatgccgttcaccaacttcaacagtattaagtgcgatggcgatttgttgaagttcttccggacatacttccccgatgcgattga
tctgattggtcgtgagcggttggttaaggatttctttaagaccaggcctcaatcgttggttatgatcaagtgtaagccatataatgtgggcggcaaggcggtgatcattggtgatgcggcacatgccatggttccc
ttctacgggcagggaatgaatgccggattcgaggaTTGTACTGTGTTGACCGAGTTGTTCAATCAACATGGCAGTGACGTTGATAGGATACTGGCTGAGTTTAGTGATACG
CGTTGGGAGGATGCACACTCTATCTGCGATCTGGCCATGTATAATTATGTTGAGGTTAGTATATGGTCTTTTATTTATATCGTACGTTTTGTATGCGGTCGTTTT
GTAGGTACCGTA -g gccatataatgtgggcggca,ggcggtgatcattggtgatg,ggttcccttctacgggca,CACAGTACAAtcctcgaatc -q [20 or 30] -qwc
211-254_264-285_294-316 --offset_around_cut_to_plot 80 --skip_failed
```

Modification rates of nucleotides surrounding the sgRNA recognition sites were plotted with GraphPad Prism 9. In cases of insertions, CRISPResso2 counts the nucleotides on both sides of the insertion as mutant in the output file. In this case rates were calculated using only the ‘insertion left’ dataset, to avoid counting the same mutation twice. Rates of unmodified nucleotides were calculated by simple subtraction (1 - modification rate) and subsequently plotted with GraphPad Prism 9.
Phenotype data analysis
We carried out all phenotype analyses using R version 3.6.2 (R Development Core Team) (62). Data sets were summarized using ‘tidyverse’ (63) and figures generated using ‘ggplot2’ (64). Likelihood ratio tests carried out with ‘DescTools’ (65). Generalized linear mixed model analyses were carried out using ‘lme4’ (66), and summarized with ‘emmeans’ (67) and ‘sjPlot’ (68), model residuals were checked for

violations of assumptions using the 'DHARMa' package (https://github.com/Philip-Leftwich/Population-level-demonstration-of-multiplex-drive-Aedes-aegypti) (69).

For manuscripts utilizing custom algorithms or software that are central to the research but not yet described in published literature, software must be made available to editors and reviewers. We strongly encourage code deposition in a community repository (e.g. GitHub). See the Nature Portfolio guidelines for submitting code & software for further information.

Data

Policy information about availability of data

All manuscripts must include a data availability statement. This statement should provide the following information, where applicable:

- Accession codes, unique identifiers, or web links for publicly available datasets
- A description of any restrictions on data availability
- For clinical datasets or third party data, please ensure that the statement adheres to our policy

All raw reads from amplicon-Seq generated in this study were submitted to NCBI SRA with the accession number PRJNA741076 (https://www.ncbi.nlm.nih.gov/bioproject/PRJNA741076/). Addgene plasmid # 52891; http://n2t.net/addgene:52891; RRID:Addgene_52891. Plasmid sequences are available from NCBI OP728003 https://www.ncbi.nlm.nih.gov/nuccore/OP728003, OP728004 {https://www.ncbi.nlm.nih.gov/nuccore/OP728004, OP728005 https://www.ncbi.nlm.nih.gov/nuccore/OP728005. The remaining data generated for this study is available in the supplemental dataset 1 file and source data are provided with this paper.

Research involving human participants, their data, or biological material

Policy information about studies with human participants or human data. See also policy information about sex, gender (identity/presentation), and sexual orientation and race, ethnicity and racism.

Reporting on sex and gender	Not applicable
Reporting on race, ethnicity, or other socially relevant groupings	Not applicable
Population characteristics	Not applicable
Recruitment	Not applicable
Ethics oversight	Not applicable

Note that full information on the approval of the study protocol must also be provided in the manuscript.

Field-specific reporting

Please select the one below that is the best fit for your research. If you are not sure, read the appropriate sections before making your selection.

☒ Life sciences ☐ Behavioural & social sciences ☐ Ecological, evolutionary & environmental sciences

For a reference copy of the document with all sections, see nature.com/documents/nr-reporting-summary-flat.pdf

Life sciences study design

All studies must disclose on these points even when the disclosure is negative.

Sample size	A basic power analysis was performed and determined that for 0.8 power, we should analyse at least 200 for a 10% difference between inheritance rates.
Data exclusions	No data were excluded from analysis.
Replication	kmosgRNAs adult females and males (at least 20) were crossed to the opposite sex bgcn-Cas9 adults to generate trans-heterozygous adults (F1) with each mating event representing a biological replicate and all replicates were successful. For initial assessments of kmosgRNAs transgene inheritance, F1 adults were pooled into groups of at least 5 trans-heterozygous females or males and crossed to WT. All F1 trans-heterozygotes displayed a mosaic eyed phenotype. All progeny (F2) were screened for presence of each transgene and eye color phenotype. Multiple replicate F1 of each genotype/phenotype were randomly selected for each cross. No replicates were excluded.
Randomization	F1 mosquitoes were screened for presence of the fluorescent markers indicating each transgene and separated by sex for mating. Trans-heterozygotes were randomly selected for crossing and all their F2 progeny were scored.
Blinding	Blinding was not performed as none of the data collected were subjective.

Reporting for specific materials, systems and methods

We require information from authors about some types of materials, experimental systems and methods used in many studies. Here, indicate whether each material, system or method listed is relevant to your study. If you are not sure if a list item applies to your research, read the appropriate section before selecting a response.

Materials & experimental systems

n/a	Involved in the study
☒	☐ Antibodies
☒	☐ Eukaryotic cell lines
☒	☐ Palaeontology and archaeology
☐	☒ Animals and other organisms
☒	☐ Clinical data
☒	☐ Dual use research of concern
☒	☐ Plants

Methods

n/a	Involved in the study
☒	☐ ChIP-seq
☒	☐ Flow cytometry
☒	☐ MRI-based neuroimaging

Animals and other research organisms

Policy information about studies involving animals; ARRIVE guidelines recommended for reporting animal research, and Sex and Gender in Research

Laboratory animals	Aedes aegypti Liverpool strain was used, adults were crossed when 2-3 days post eclosion and allowed to mate for at least 2 days prior to bloodfeeding. Fluorescent screening was performed on L3/L4 larvae or pupae.
Wild animals	Wild animals were not used in this study.
Reporting on sex	Data was collected and is reported separately for each sex of Cas9 bearing F0/F1.
Field-collected samples	No field collected samples were used in this study.
Ethics oversight	No ethical oversight is required for mosquitoes, however this work was approved by The Biological and Genetic Modification Safety Committee.

Note that full information on the approval of the study protocol must also be provided in the manuscript.
