# Supplementary material for: A multiplexed, confinable CRISPR/Cas9 gene drive can propagate in caged Aedes aegypti populations
Source: Nat Commun. 2024 Jan 25;15:729. doi: 10.1038/s41467-024-44956-2 (PMC10810878; doi:10.1038/s41467-024-44956-2)
Supplement: Supplementary file 4 — Description of Additional Supplementary Files [file 41467_2024_44956_MOESM4_ESM.pdf]

Description of Additional Supplementary Files

Legend of Dataset S1:

Cage trial screening. Screening results for each generation of the cage trial. F1 to F6 progeny results classified by eye phenotype (DE,dark eyes; ME, mosaic eyes; WE, white eyes) for the multi-generational cage trial. TH (trans-heterozygous, *kmo*^{sgRNAs};*bgcn*-Cas9D).

Eye phenotype. Number of individuals with mosaic/white eye phenotype in *kmo*^{sgRNAs} and non-*kmo*^{sgRNAs} mosquitoes in each generation. *kmo*^{sgRNAs} = all progeny that inherited the *kmo*^{sgRNAs} element (*kmo*^{sgRNAs} only as well as trans-heterozygotes); Non-*kmo*^{sgRNAs} = progeny that did not inherit the *kmo*^{sgRNAs} element (*bgcn*-Cas9 only and wild type).

Genotype. Phenotype frequencies for each generation of the cage trial assay. Phenotype frequencies calculated from the F1-F6 generations (Number of mosquitoes showing a phenotype/total mosquitoes screened). TH: trans-heterozygous; WT: wild type.

Larvae. Number of larvae estimated in each experimental cage from F1 to F6.
